# Greater sage‐grouse respond positively to intensive post‐fire restoration treatments

**DOI:** 10.1002/ece3.8671

**Published:** 2022-03-21

**Authors:** Sharon A. Poessel, David M. Barnard, Cara Applestein, Matthew J. Germino, Ethan A. Ellsworth, Don Major, Ann Moser, Todd E. Katzner

**Affiliations:** ^1^ U.S. Geological Survey, Forest and Rangeland Ecosystem Science Center Boise Idaho USA; ^2^ Agricultural Research Service U.S. Department of Agriculture Water Management and Systems Research Fort Collins Colorado USA; ^3^ Bureau of Land Management Boise Idaho USA; ^4^ 115870 Idaho Department of Fish and Game Boise Idaho USA

**Keywords:** *Centrocercus urophasianus*, cheatgrass, disturbance, resource selection function, sagebrush, wildfire

## Abstract

Habitat loss is the most prevalent threat to biodiversity in North America. One of the most threatened landscapes in the United States is the sagebrush (*Artemisia* spp.) ecosystem, much of which has been fragmented or converted to non‐native grasslands via the cheatgrass‐fire cycle. Like many sagebrush obligates, greater sage‐grouse (*Centrocercus urophasianus*) depend upon sagebrush for food and cover and are affected by changes to this ecosystem. We investigated habitat selection by 28 male greater sage‐grouse during each of 3 years after a 113,000‐ha wildfire in a sagebrush steppe ecosystem in Idaho and Oregon. During the study period, seeding and herbicide treatments were applied for habitat restoration. We evaluated sage‐grouse responses to vegetation and post‐fire restoration treatments. Throughout the 3 years post‐fire, sage‐grouse avoided areas with high exotic annual grass cover but selected strongly for recovering sagebrush and moderately strongly for perennial grasses. By the third year post‐fire, they preferred high‐density sagebrush, especially in winter when sagebrush is the primary component of the sage‐grouse diet. Sage‐grouse preferred forb habitat immediately post‐fire, especially in summer, but this selection preference was less strong in later years. They also selected areas that were intensively treated with herbicide and seeded with sagebrush, grasses, and forbs, although these responses varied with time since treatment. Wildfire can have severe consequences for sagebrush‐obligate species due to loss of large sagebrush plants used for food and for protection from predators and thermal extremes. Our results show that management efforts, including herbicide application and seeding of plants, directed at controlling exotic annual grasses after a wildfire can positively affect habitat selection by sage‐grouse.

## Graphical abstract

We investigated habitat selection by 28 male greater sage‐grouse during each of 3 years after a wildfire in Idaho and Oregon. Sage‐grouse avoided areas with high exotic annual grass cover but selected for recovering sagebrush, areas that were intensively treated with herbicide, and areas that were seeded with sagebrush, grasses, and forbs. Our results show that management efforts directed at controlling exotic annual grasses after a wildfire can positively affect habitat selection by sage‐grouse.

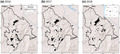

## 1 INTRODUCTION

The most prevalent threat to biodiversity in North America is habitat loss (Venter et al., 2006; Wilcove et al., 1998). Habitat fragmentation and loss can be caused by natural or anthropogenic activities, including wildfire, agriculture, urban development, outdoor recreation, and extractive land uses (Wilcove et al., 1998). Furthermore, synergies between multiple stressors can exacerbate the effects of habitat loss. For example, increasing urbanization can alter the fire regime of natural habitats, resulting in significant consequences to biodiversity (Regan et al., 2010).

One of the most threatened landscapes in the United States is the sagebrush (*Artemisia* spp.) ecosystem (Davies et al., 2011; Knick et al., 2003; Wisdom et al., 2005). Wildfire is a primary threat to sagebrush communities, particularly in cases where post‐fire landscapes are invaded by exotic annual grasses, including cheatgrass (*Bromus tectorum*) and medusahead (*Taeniatherum caput*‐*medusae*; Pellant & Hall, 1994). Cheatgrass has become the most prolific invasive plant species in the northern Great Basin (Boyte et al., 2016), and >30% of land in the Intermountain West is estimated to have a high abundance (≥15% cover) of cheatgrass (Bradley et al., 2018). Additionally, cheatgrass can alter the functions of sagebrush ecosystems through positive feedback mechanisms that cause an increase in the frequency and extent of wildfires (Germino et al., 2016). For example, in the Intermountain West, the presence of cheatgrass can double the frequency of fires (Bradley et al., 2018).

Management strategies for reducing the threat of invasive species in sagebrush steppe include application of herbicides such as imazapic, which can reduce the germination of annuals for up to 1–2 years, or seeding of sagebrush, grasses, and forbs (Davies et al., 2011). These treatments often are administered after wildfire to promote assembly of desirable perennials over exotic annuals (Monaco et al., 2016). The success of post‐fire treatments in sagebrush steppe has been mixed at best and may be influenced by elevation, climate, or topography (Arkle et al., 2014; Knutson et al., 2014). However, newer strategies for treatment application and more precise assessment of outcomes in space and time appear to be demonstrating greater success (Applestein, Germino, & Fisk, 2018; Germino et al., 2018; O'Connor et al., 2020).

Despite restoration interventions, wildfires are one of many stressors increasing fragmentation of sagebrush ecosystems, contributing to declines and, in some cases, extirpations of sagebrush‐associated wildlife species (Davies et al., 2011). For example, loss of populations of sage‐grouse (*Centrocercus* spp.) is significantly related to loss of sagebrush habitat (Aldridge et al., 2008; Coates et al., 2016). Likewise, abundance and distribution of other sagebrush‐obligate wildlife, some of which are of special conservation concern, are sensitive to multi‐scale habitat changes, including fragmentation and loss of shrublands (Katzner & Parker, 1997; Knick & Rotenberry, 2000, 2002; Shipley et al., 2006).

The greater sage‐grouse (*C. urophasianus*; hereafter, “sage‐grouse”) is a sagebrush‐obligate species of conservation interest that has been viewed as a possible umbrella species (Hanser & Knick, 2011; Rowland et al., 2006). Sage‐grouse distributions in North America have shrunk more than 40% from their historical range, primarily due to degradation and conversion of sagebrush habitat (Schroeder et al., 2004). During winter, sage‐grouse use sagebrush of several species for food (>99% of the diet), but they have a more diverse diet in summer that also includes forbs and insects, and a transitional diet in spring and fall (Gregg et al., 2008; Patterson, 1952; Remington & Braun, 1985; Wallestad et al., 1975). Fires can be detrimental to sage‐grouse because, although forbs usually recover quickly after a fire (i.e., within as little as 1–2 years), sagebrush sometimes can take 30 years or more to return to pre‐fire conditions, if it does at all (Beck et al., 2009; Rhodes et al., 2010; Shriver et al., 2019). Thus, many years may pass before post‐burn winter habitat conditions meet sage‐grouse requirements for food or cover (Beck et al., 2009; Fremgen‐Tarantino et al., 2020). Not surprisingly, sage‐grouse survival, recruitment, and productivity can decline in the years following large fires (Blomberg et al., 2012; Foster et al., 2019).

Although sage‐grouse response to fire has been investigated previously (e.g., Anthony et al., 2021; Lockyer et al., 2015), little information is available about how post‐fire restoration treatments affect patterns in resource selection by sage‐grouse. To address this information gap, we used telemetry data to evaluate habitat selection by sage‐grouse in each of 3 years after a large wildfire in sagebrush steppe in the Intermountain West. Because landscape conditions prior to a disturbance can influence post‐disturbance responses of wildlife (Knick & Rotenberry, 2000), and because we expected that sage‐grouse might use the same areas post‐fire as they did before the fire (Foster et al., 2019), we considered pre‐fire land cover data in our analyses. We predicted that sage‐grouse would avoid landscapes dominated by exotic annual grasses because these areas lacked sufficient food and cover. We also predicted that sage‐grouse would select for: (1) areas of recovering sagebrush, especially in winter when these shrubs could provide food and cover from predators, (2) landscapes with recovering forbs, primarily in summer when these forbs may be a food resource, and (3) tall perennial bunchgrasses that could provide cover year‐round. We evaluated these predictions in three ways, as a response to pre‐fire land cover, to post‐fire vegetation, and to post‐fire treatments applied to the landscape. Our study provides insight into the behavior of sage‐grouse and unique information on their response to intensive restoration efforts after a large‐scale disturbance on the landscape.

## 2 METHODS

### 2.1 Study area, sage‐grouse capture, and telemetry

**FIGURE 1:**
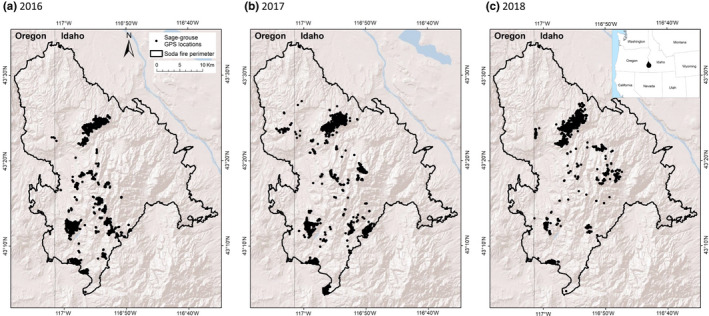
Map of the Soda Wildfire study area and telemetry locations from 28 sage‐grouse in southern Idaho and Oregon in each of three years, (a) 2016 (10 birds), (b) 2017 (16 birds), and (c) 2018 (10 birds). Map projection is UTM Zone 11N

We considered sage‐grouse data collected inside the perimeter of the Soda Wildfire along the southwestern Idaho‐southeastern Oregon border (Figure 1). This fire burned 113,000 ha, including 111,000 ha of designated sage‐grouse habitat, in August 2015. During the 2015 breeding season prior to the fire, 10 leks were "active" (i.e., had >1 male sage‐grouse displaying) in the study area. Elevation in the study area ranges from 750 to 2055 m above sea level (Germino et al., 2018). Climate is typical of arid basin and range topography, with dry, hot summers and wet, cold winters. Sagebrush steppe is by far the most abundant historical plant community type across the study area. Here, that habitat consists primarily of Wyoming big sagebrush (*Artemisia tridentata* ssp. *wyomingensis*), with lesser amounts of basin big sagebrush (*A. t*. ssp. *tridentata*) and low sagebrush (*A. arbuscula*). The lower elevation limit for sagebrush steppe occurs along the eastern, northern, and northwestern boundaries of the burned area, where fire and exotic annual grasses have degraded the dominant salt desert habitat. The landscape has a long history of livestock utilization, which, in recent decades, has consisted of cattle (*Bos taurus*) and sheep (*Ovis aries*) grazing in spring or fall. Additional details of the wildfire and study area are described elsewhere (Applestein, Germino, & Fisk, 2018; Applestein, Germino, Pilliod, et al., 2018; Germino et al., 2018, 2019).

We captured male sage‐grouse near known leks in the study area using spotlighting and netting techniques at night (Giesen et al., 1982) during March–May over a 3‐year period, from 2016 to 2018. We determined the sex of sage‐grouse based on physical characteristics and fitted each bird with a 22‐g Argos/GPS solar‐powered Platform Transmitter Terminal (Microwave Telemetry, Inc., Columbia, MD, USA) using a rump‐mount technique (Wann et al., 2019). Telemetry units generally collected five to six locations per day, one to two of which were at night. Animal capture and handling protocols were approved by the Idaho Department of Fish and Game (IDFG).

### 2.2 Telemetry data processing

For analysis, we excluded GPS data collected from sage‐grouse that wandered outside of the perimeter of the Soda Wildfire. We further limited GPS data by excluding portions of the study area that a previous model identified as being unsuitable for sage‐grouse, based on telemetry, observation data, and environmental parameters (IDFG, 2019). We also excluded data that were collected from a bird with <10 locations or <10 days of data.

Each remaining location was collected in one of three years, 2016, 2017, or 2018, and in one of four seasons that we defined based on the local sage‐grouse breeding cycle: winter (1 December–14 February), breeding (15 February–15 May), summer (16 May–31 August), and fall (1 September–30 November). Because each winter season incorporated a portion of two years, we grouped December locations with those from the following year (e.g., December 2016 locations were categorized as 2017 locations). Finally, we removed all nighttime locations because we presumed that habitat selection by sage‐grouse might differ between day and night. In winter, we defined nighttime locations as those between sunset and sunrise. In all other seasons, we defined nighttime locations as those between sunset and 1 h before sunrise.

### 2.3 Environmental data

We evaluated sage‐grouse habitat use in the context of three types of environmental data: pre‐fire land cover, post‐fire vegetation, and post‐fire treatments. The first of these were 2012 (pre‐fire) land cover data derived from the Landscape Fire and Resource Management Planning Tools Project (LANDFIRE; Rollins, 2009). We used ArcGIS v.10.7 (Esri, Redlands, CA) to condense this 30‐m resolution dataset into six pre‐fire land cover types, as follows: big sagebrush shrubland (hereafter, “big sagebrush”; 37.9% of the study area), low sagebrush shrubland (hereafter, “low sagebrush”; 18.7%), introduced annual grassland (16.4%), grassland and steppe (consisting of large, extensive grasslands with no or low shrub cover; 12.4%), trees (8.9%), and "other" (5.7%).

The second type of environmental data were maps interpolated from post‐fire vegetation data collected in each year of the study (2016–2018, combining certain methods from Applestein, Germino, & Fisk, 2018; Applestein, Germino, Pilliod, et al., 2018; Germino et al., 2018, 2019). We collected field sampling data annually on density of all sagebrush species, and on cover of exotic annual grasses, forbs, Sandberg bluegrass (*Poa secunda*), and other perennial bunchgrasses. We considered Sandberg bluegrass separately from other perennial bunchgrasses because it is smaller and matures earlier than other grasses, and thus may be relevant to sage‐grouse during times of the year when other grasses are not available (Davies et al., 2014, 2015). We selected plots with stratified random sampling with a density of one plot per 54.5 ha (number of plots in each year ranged from 1361 to 2135; Applestein, Germino, & Fisk, 2018; Applestein, Germino, Pilliod, et al., 2018).

We collected cover data from a 3 × 2 m overhead photo taken at 2‐m nadir over plot centers and, in 2017 and 2018, a second 3 × 2 m photo taken at 5.5 m directly south of plot centers. In 2017 and 2018, when plants had become taller, we cropped the photos to retain only the center 50% area to reduce parallax effects around the edges. We then assessed cover of individual species using SamplePoint (Booth et al., 2006), a grid‐point intercept software, with 49 (in 2017 and 2018) or 100 (in 2016) points per photo. We categorized each species into a functional group and obtained total percent cover of each functional group.

To estimate sagebrush density, we conducted frequency‐density monitoring, where we first searched a 1‐m quadrat at plot centers for sagebrush seedlings. If we found fewer than three individual seedlings, we expanded our search area to circles of increasing radii (i.e., 5.5, 9, 13, and 18 m) until we found at least three seedlings. We then counted the total number of sagebrush seedlings and remnants in that plot. We calculated density as the total number of individuals divided by the area searched, then scaled to ha. We then binned sagebrush density into the following four categories for analysis: (1) 0 plants/ha, (2) 1–100 plants/ha, (3) 101–1000 plants/ha, and (4) >1000 plants/ha.

We created interpolated vegetation cover and sagebrush density maps of the Soda Wildfire via a two‐step process. First, we ran a random forest analysis (“caret” package; Kuhn, 2008) in R (R Core Team, 2017) that predicted, for each 30‐m pixel, each vegetation cover or density using the following landscape covariates: elevation (m; USGS, 2015), aspect, slope, % fertile islands (areas where shrubs existed pre‐fire that had enhanced hydrologic and biogeochemical soil properties, appearing post‐fire as darkened soil with high organic content), and soil characteristics from the Soil Survey Geographic Database (USDA NRCS, 2020), including cation exchange, percent clay, coarse fragments, percent organic content, and percent sand. Second, we predicted vegetation cover across the continuous area of the Soda Wildfire by kriging the residuals of the random forest model (“gstat” package in R; Pebesma, 2004). Because residuals from binned data cannot be kriged, we did not krige residuals for the sagebrush density maps.

The third type of environmental data were spatially explicit information on post‐fire restoration treatments applied within the study area. The U.S. Department of Interior implements an Emergency Stabilization and Rehabilitation (ESR) program to restore areas damaged by wildfire. The Soda Wildfire ESR included restoration treatments for the three years post‐fire, although treatments were applied at different times and over different extents of the burned area. Aerial sagebrush/forb seeding, which consisted of seed mixes of three naturalized forbs (alfalfa [*Medicago sativa*], small burnet [*Sanguisorba minor*], and yarrow [*Achillea millefolium*]) and sagebrush (either Wyoming big sagebrush, basin big sagebrush, or low sagebrush), was applied in winter 2016 over 61,450 ha and in winter 2017 over 6855 ha. Aerial sagebrush/grass seeding, which consisted of Sandberg bluegrass and big sagebrush, was applied in winter 2018 over 9575 ha. Aerial forb seeding, which consisted of a species‐rich mix of forbs known to be preferred by sage‐grouse, was applied in winter 2016 over 2885 ha and in winter 2017 over 2420 ha. Drill seeding, which consisted of a mix of native and introduced grass species, was applied in fall 2015 over 7230 ha, in fall 2016 over 4620 ha, and in fall 2017 over 860 ha. Finally, pre‐emergent herbicide (imazapic) was applied in fall 2015/winter 2016 (hereafter, fall 2015) over 10,940 ha and in fall 2016 over 25,350 ha. In many cases, treatment coverages overlapped.

Areas treated with drill seeding or herbicide application were mid‐elevation sites that were susceptible to cheatgrass invasion but where perennial grasses were more likely to establish (Chambers et al., 2014). Drill seeding was also focused on areas that were less rocky and that were accessible to tractors. Areas that were aerially seeded with grasses typically had rougher terrain that was not accessible to drill seeding equipment. Almost all of the burned land managed by the U.S. Bureau of Land Management (BLM) in Idaho was seeded with the sagebrush/forb mix, but none was seeded in Oregon. Finally, areas that were aerially seeded with forbs were designed to include sage‐grouse lek sites, although BLM assessments indicated little establishment from these seeds. These treatments are described in further detail elsewhere (Applestein, Germino, & Fisk, 2018; Applestein, Germino, Pilliod, et al., 2018; Germino et al., 2018, 2019).

### 2.4 Characterizing habitat features in resource selection functions

We used resource selection functions to investigate sage‐grouse use, relative to availability, of habitat within the study area (Johnson, 1980; Manly et al., 2002). To do this, we identified areas that sage‐grouse used and areas that were available to them to be used. The average daily distance traveled by an individual sage‐grouse in our study was ~990 m; thus, we assumed that responses of sage‐grouse to habitat would be roughly within a circular area with a diameter of 1 km.

Consequently, we quantified attributes of habitat that sage‐grouse used within 500‐m radius buffers ("use buffers") around each sage‐grouse telemetry location, removing any part of the buffer that extended outside of the modeled sage‐grouse habitat within the Soda Wildfire perimeter. For each use buffer, we determined (1) the mean percent cover of each of the four post‐fire vegetation cover types, and the proportion of the buffer (2) composed of each sagebrush density bin, (3) composed of each pre‐fire land cover type, and (4) to which each post‐fire restoration treatment was applied. We also determined these characteristics for 500‐m buffers around random points available to sage‐grouse (“available buffers”). To do this, we first created, for each bird in each year and each season, a polygon with a 5‐km radius around all telemetry points, excluding area outside the modeled sage‐grouse habitat within the Soda Wildfire perimeter. Within each polygon, we then generated a set of random points that equaled the number of sage‐grouse telemetry points.

### 2.5 Statistical analysis

We built resource selection functions with generalized linear mixed‐effects models (“lme4” package; Bates et al., 2015) in R (R Core Team, 2018). Our response variable was binary, with “1” representing the covariates associated with the use buffers and “0” representing the covariates associated with the available buffers (Manly et al., 2002). We built model sets for each of our three types of environmental data—one for the pre‐fire land cover data, one for the post‐fire vegetation data, and one for the post‐fire treatment data. For each type of environmental data, we ran three sets of models, one for each year of the study (2016–2018). Because we were interested in evaluating seasonal variation in selection of habitat by sage‐grouse, the pre‐fire land cover and post‐fire vegetation models included two‐way interactions between season and each habitat variable. The models describing post‐fire treatments had large numbers of predictors. Thus, to reduce the overall number of parameters, we included season only as a single fixed effect in these models (i.e., we did not model interactions).

In all models, we included the individual sage‐grouse ID as a random effect to account for repeated measurements of individuals. We rescaled the continuous predictor variables by subtracting the mean and dividing by two times the standard deviation (Gelman, 2008). We tested correlations between pairs of variables in each model, and we removed one of the variables in any pair that had a correlation ≥0.60 (a conservative threshold; Dormann et al., 2013). We suspected that the correlated variables we excluded from models could have been important to sage‐grouse (e.g., Sveum et al., 1998). To account for this, for each type of environmental data and for each year, we ran one set of models to evaluate responses to uncorrelated variables and another set to evaluate responses to the previously excluded correlated variables. We describe the models with the correlated variables in Appendix S1 (Additional Methods), although we present results from these models in the main text because they were useful for inference.

To evaluate sage‐grouse response to pre‐fire land cover, we initially ran two submodels for each of the three years of the study. Each submodel had four fixed effects. The first three fixed effects were the same in both submodels and described the interactions between season and cover of each of introduced annual grassland, trees, and grassland and steppe. The fourth fixed effect described the interaction between season and cover of either big sagebrush (one submodel) or low sagebrush (second submodel). We chose this approach because cover of big sagebrush was negatively correlated with that of low sagebrush; thus, both could not be included in the same model. We then identified, for each year, the submodel with the lower Akaike's Information Criterion corrected for small sample size (AICc) value, and we carried only that model forward for subsequent analysis.

To evaluate sage‐grouse response to post‐fire vegetation, our three models, one for each year, also included four fixed effects. These described four interactions, one each between season and cover of exotic annual grasses, forbs, perennial bunchgrasses, and Sandberg bluegrass. We excluded sagebrush density from these models because three of the density bins were correlated with perennial bunchgrass cover (also see Germino et al., 2018).

To evaluate sage‐grouse response to post‐fire treatments, we built model sets of uncorrelated variables describing the coverage of treatments applied prior to the year of the sage‐grouse data collection. The model for 2016 included four fixed effects, the model for 2017 included six fixed effects, and the model for 2018 included eight fixed effects. Each model included, as fixed effects, sage‐grouse season and aerial seeding of sagebrush/forbs and of forbs, both conducted in winter 2016. The 2016 model also included a fixed effect for application of herbicides in fall 2015. The 2017 model also included fixed effects for aerial seeding of sagebrush/forbs and of forbs, both conducted in winter 2017, and for application of herbicides in fall 2016. Finally, the 2018 model also included fixed effects for aerial seeding of sagebrush/forbs and of forbs, both conducted in winter 2017, for aerial seeding of sagebrush/grasses conducted in winter 2018, and for drill seeding conducted in fall 2015. In this model, we also included slope (derived from a 30‐m resolution digital elevation model; USGS, 2015) as an eighth fixed effect because drill seeding was only applied in areas with <40° slope (Germino et al., 2018).

Our modeling approach resulted in nine primary models with uncorrelated variables (one model for each of the three years and for each of the three types of environmental data) and an additional 10 models with correlated variables (Appendix S1, Additional Methods). For each of these 19 final models, we used the dredge function in the “MuMIn” R package (Bartoń, 2018) to evaluate all possible submodels (range = 4–256 submodels per model; Doherty et al., 2012), and we used AICc to rank the submodels (Anderson, 2008; Burnham & Anderson, 2002). When the top‐ranked submodel had <95% weight, we averaged the submodels with weights ≥0.01. We based our interpretations on either the top‐ranked submodel (when it had >95% weight) or the averaged submodels (when the top‐ranked submodel had <95% weight). Finally, we used the “effects” R package (Fox & Weisberg, 2019) to construct plots examining the probability of sage‐grouse use of each predictor variable for the top‐ranked model.

## 3 RESULTS

We collected 39,813 telemetry locations from 41 sage‐grouse over the 3‐year study period. After removing sparse data, nighttime locations, and telemetry locations outside the study area, we used 16,273 locations ($\bar{x}$ ± SD = 581 ± 671 locations per bird; range = 35–2905 locations) collected from April 2016 to November 2018 (143 ± 169 number of days of data per bird; range = 12–712 days) from 28 sage‐grouse. This included 4333 locations from 10 birds in 2016, 6886 locations from 16 birds in 2017, and 5054 locations from 10 birds in 2018.

The average percentage of perennial bunchgrass cover at sage‐grouse locations increased throughout the study (from 7.5% cover in 2016, to 18.0% in 2017 and 25.2% in 2018). In general, the other vegetation cover types at sage‐grouse locations remained relatively constant across study years, with the average percent cover ranging from 10% to 14% for exotic annual grasses, from 1% to 2.5% for forbs, and from 13% to 16% for Sandberg bluegrass over the three years. In 2016, over 93% of sage‐grouse locations were in areas with no sagebrush and only 3.6% were in areas with sagebrush density >100 plants/ha. However, by 2018, only 33% were in areas with no sagebrush and almost 64% were in areas with sagebrush density >100 plants/ha.

### 3.1 Habitat selection

In nearly all cases, for analyses of sage‐grouse response to pre‐fire land cover, post‐fire vegetation, and post‐fire treatments, the full (global) model had the majority of model weights (i.e., the most support; Table 1). The one exception was during the first year of the study (2016) for the pre‐fire land cover model. We used model‐averaging for three of the 19 top‐ranked models that had <95% weight.

**TABLE 1:** Highest‐ranked linear mixed‐effects models used to evaluate greater sage‐grouse resource selection of pre‐fire land cover, post‐fire vegetation, and post‐fire treatments in the Soda Wildfire area in southern Idaho and Oregon, 2016–2018

Year	Model group	Model description	*w*
**2016**	*Pre‐fire land cover:*		
	Uncorrelated variables	Introduced annual grassland (IAG) + season*big sagebrush shrubland (BS) + season*grassland and steppe (GS) + season*trees	0.67
	Correlated variables	Season*low sagebrush shrubland (LS)	0.96
	*Post‐fire vegetation:*		
	Uncorrelated variables	Season*exotic annual grass (EAG) + season*forb + season*perennial bunchgrass (PB) + season*Sandberg bluegrass (SB)	1.00
	*Post‐fire treatments:*		
	Uncorrelated variables	Season + aerial seeding of sagebrush/forbs (SS/F; winter 2016) + aerial seeding of forbs (SF; winter 2016) + herbicide application (HERB; fall 2015)	1.00
	Correlated variables	Season + drill seeding (DRILL; fall 2015) + slope	0.90
**2017**	*Pre‐fire land cover:*		
	Uncorrelated variables	Season*BS + season*IAG + season*GS + season*trees	0.99
	Correlated variables	Season*LS	0.92
	*Post‐fire vegetation:*		
	Uncorrelated variables	Season*EAG + season*forb + season*PB + season*SB	1.00
	Correlated variables	Season*sagebrush density 1–100 plants/ha (bin 2) + season*sagebrush density 101–1000 plants/ha (bin 3) + season*sagebrush density > 1000 plants/ha (bin 4)	1.00
	*Post‐fire treatments:*		
	Uncorrelated variables	Season + SS/F (winter 2016) + SS/F (winter 2017) + SF (winter 2016) + SF (winter 2017) + HERB (fall 2016)	1.00
	Correlated variables	Season + DRILL (fall 2015) + DRILL (fall 2016) + slope	1.00
	Correlated variables	Season + HERB (fall 2015)	1.00
**2018**	*Pre‐fire land cover:*		
	Uncorrelated variables	Season*LS + season*IAG + season*GS + season*trees	1.00
	Correlated variables	Season*BS	1.00
	*Post‐fire vegetation:*		
	Uncorrelated variables	Season*EAG + season*forb + season*PB + season*SB	1.00
	Correlated variables	Season*sagebrush density bin 2 + season*sagebrush density bin 3 + season*sagebrush density bin 4	1.00
	*Post‐fire treatments:*		
	Uncorrelated variables	Season + SS/F (winter 2016) + SS/F (winter 2017) + SF (winter 2016) + SF (winter 2017) + aerial seeding of sagebrush/grasses (winter 2018) + DRILL (fall 2015) + slope	0.95
	Correlated variables	Season + DRILL (fall 2016) + DRILL (fall 2017) + slope	1.00
	Correlated variables	Season + HERB (fall 2015) + HERB (fall 2016)	1.00

Note

If the highest‐ranked model had a weight <0.95, we averaged all subset models with weights ≥0.01.

Abbreviation: *w*, model weight.

**FIGURE 2:**
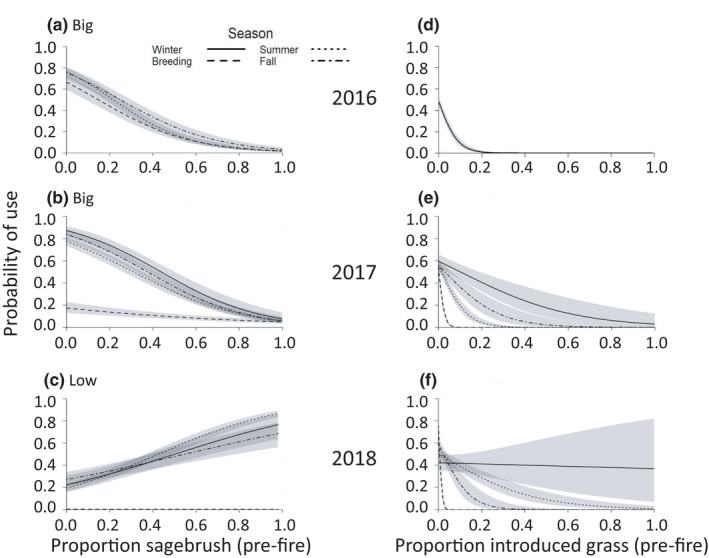
Probability of use by greater sage‐grouse, by season, year (2016–2018), and proportion of 500‐m buffers around use and available points that were composed of pre‐fire land cover types, in the Soda Wildfire study area. Plots show response to pre‐fire big sagebrush cover by sage‐grouse in (a) 2016 and (b) 2017, to pre‐fire low sagebrush cover by sage‐grouse in (c) 2018, and to pre‐fire introduced grasslands by sage‐grouse in (d) 2016 (across seasons; highest‐ranked model did not include the interaction), (e) 2017, and (f) 2018. Winter season was not represented in 2016. Gray bands represent 95% confidence intervals

The influence of pre‐fire features on habitat selection by sage‐grouse varied among the years of the study. The suite of variables in the three top models describing sage‐grouse response to pre‐fire land cover included areas mapped pre‐fire as big sagebrush for sage‐grouse data in 2016 and 2017, and areas mapped pre‐fire as low sagebrush for sage‐grouse data in 2018. In all seasons and years, based on pre‐fire mapping, sage‐grouse were less likely to use areas of big sagebrush and more likely to use areas of low sagebrush (Figure 2a‐c, Figure S1; Table 2a, Tables S1–S6; beta estimates, *SE*s, *z*‐values, and confidence intervals are provided in the Appendix S1 tables). However, in the 2018 breeding season, sage‐grouse began to increase their use of areas mapped pre‐fire as big sagebrush and correspondingly decreased their use of areas in low sagebrush (Figure 2c, Figure S1c). They also were less likely to use areas that were mapped pre‐fire as introduced grasses or trees (Figure 2d‐f, Figure S2a‐c; Table 2a, Tables S1–S3). Sage‐grouse use of areas mapped pre‐fire as grassland and steppe habitats was more variable, with higher use in the fall season in 2016 and 2018, but less use during all other seasons and years (Figure S2d‐f; Table 2a, Tables S1–S3).

**TABLE 2:** Selection of habitat variables, including interactions with season, used in (a) pre‐fire land cover models, (b) post‐fire vegetation models, and (c) post‐fire treatment models of greater sage‐grouse resource selection in the Soda Wildfire area in southern Idaho and Oregon, 2016–2018

Variable	2016	2017	2018
(a) *Pre‐fire land cover models:*			
Big sagebrush	−	−	−
Low sagebrush	+	+	+
Introduced grassland	−	−	0
Grassland and steppe	−	−	−
Trees	−	−	−
Breeding*big sagebrush	na	+	+
Summer*big sagebrush	0	0	0
Fall*big sagebrush	0	0	+
Breeding*low sagebrush	na	−	−
Summer*low sagebrush	0	0	0
Fall*low sagebrush	−	0	0
Breeding*introduced grassland	na	−	−
Summer*introduced grassland	0	−	−
Fall*introduced grassland	0	−	−
Breeding*grassland and steppe	na	0	0
Summer*grassland and steppe	0	0	+
Fall*grassland and steppe	+	+	+
Breeding*trees	na	−	−
Summer*trees	+	−	−
Fall*trees	+	−	−
(b) *Post‐fire vegetation models:*			
Exotic annual grass cover	−	−	0
Forb cover	0	0	+
Perennial bunchgrass cover	+	−	+
Sandberg bluegrass cover	+	0	+
Sagebrush density bin 2	na	+	0
Sagebrush density bin 3	na	+	+
Sagebrush density bin 4	na	−	+
Breeding*exotic annual grass cover	na	0	0
Summer*exotic annual grass cover	+	−	−
Fall*exotic annual grass cover	+	−	−
Breeding*forb cover	na	0	−
Summer*forb cover	+	0	−
Fall*forb cover	0	−	−
Breeding*perennial bunchgrass cover	na	+	+
Summer*perennial bunchgrass cover	−	+	−
Fall*perennial bunchgrass cover	0	+	−
Breeding*Sandberg bluegrass cover	na	+	0
Summer*Sandberg bluegrass cover	0	0	−
Fall*Sandberg bluegrass cover	−	0	−
Breeding*sagebrush density bin 2	na	−	+
Summer*sagebrush density bin 2	na	−	−
Fall*sagebrush density bin 2	na	0	+
Breeding*sagebrush density bin 3	na	−	−
Summer*sagebrush density bin 3	na	−	0
Fall*sagebrush density bin 3	na	−	0
Breeding*sagebrush density bin 4	na	+	0
Summer*sagebrush density bin 4	na	+	−
Fall*sagebrush density bin 4	na	+	−
(c) *Post‐fire treatment models*:			
Sagebrush/forb seeding‐winter 2016	−	−	+
Sagebrush/forb seeding‐winter 2017	na	+	+
Forb seeding‐winter 2016	+	+	+
Forb seeding‐winter 2017	na	+	+
Sagebrush/grass seeding‐winter 2018	na	na	+
Herbicide application‐fall 2015	+	+	+
Herbicide application‐fall 2016	na	+	+
Drill seeding‐fall 2015	−	−	−
Drill seeding‐fall 2016	na	+	+
Drill seeding‐fall 2017	na	na	+

Note

"+" indicates the habitat type was selected, "−" indicates the habitat type was avoided, "0" indicates the habitat type was neither selected nor avoided, and "na" indicates the habitat type was not included in the model for that year. Reference variable for season was breeding in 2016 and winter in 2017 and 2018. Sagebrush density bin 2 was 1–100 sagebrush plants/ha, bin 3 was 101–1000 sagebrush plants/ha, and bin 4 was >1000 sagebrush plants/ha.

**FIGURE 3:**
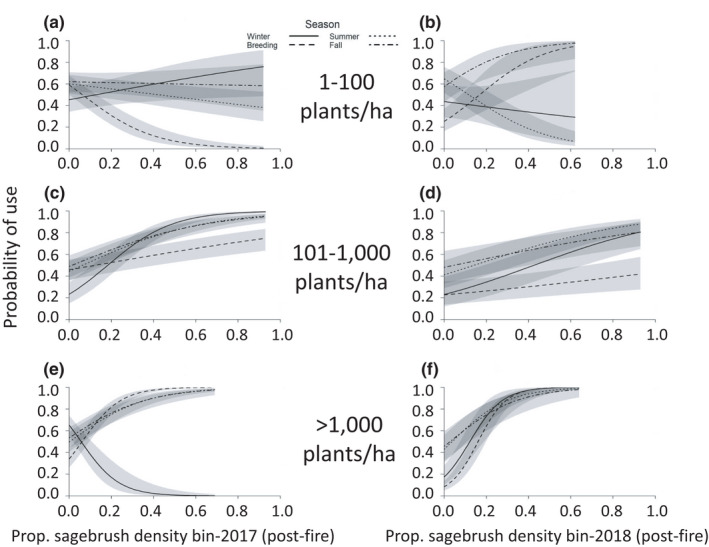
Probability of use by greater sage‐grouse, by season, year (2017–2018), and proportion of 500‐m buffers around use and available points that were composed of sagebrush density bins, in the Soda Wildfire study area. Plots show response by sage‐grouse to post‐fire sagebrush with a density of 1–100 plants/ha in (a) 2017 and (b) 2018, of 101–1000 plants/ha in (c) 2017 and (d) 2018, and of >1000 plants/ha in (e) 2017 and (f) 2018. Gray bands represent 95% confidence intervals

Post‐fire responses by sage‐grouse also changed over time. In general, sage‐grouse generally avoided areas with greater exotic annual grass cover (Figure S3a‐c; Table 2b, Tables S7–S9) and selected for perennial bunchgrass cover (Figure S3d‐f; Table 2b, Tables S7–S9), although they did not avoid exotic annual grass cover in the breeding and winter seasons in 2018, and they selected against perennial bunchgrass cover in summer 2016 and winter, summer, and fall 2017. Sage‐grouse generally selected strongly for areas with relatively higher sagebrush density (>100 plants/ha; Figure 3; Table 2b, Tables S10–S11). Patterns in selection were less clear for areas with relatively lower sagebrush density (1–100 plants/ha; Figure 3; Table 2b, Tables S10–S11), for forb cover, which was selected for in summer 2016 but not in summers of later years (Figure S4a‐c; Table 2b, Tables S7–S9), and for Sandberg bluegrass cover (Figure S4d‐f; Table 2b, Tables S7–S9).

**FIGURE 4:**
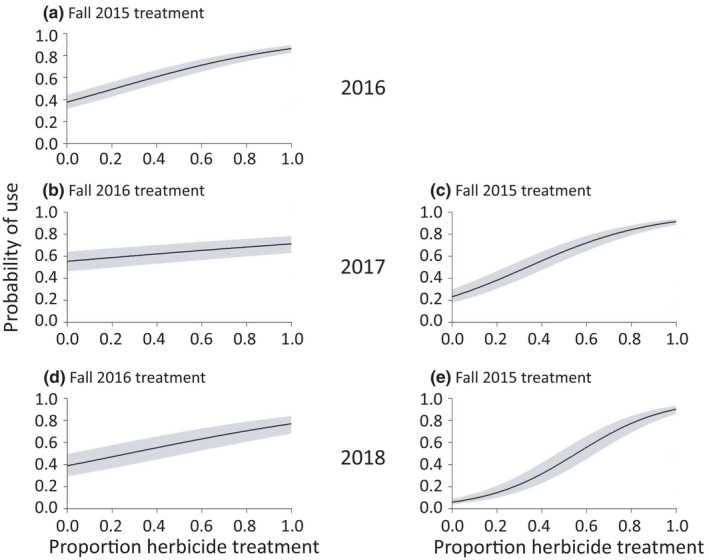
Probability of use by greater sage‐grouse, by year (2016–2018) and proportion of 500‐m buffers around use and available points that were composed of post‐fire treatment areas, in the Soda Wildfire study area. Plots show response to treatment with herbicide in (a) fall 2015 by sage‐grouse in 2016, (b) fall 2016 by sage‐grouse in 2017, (c) fall 2015 by sage‐grouse in 2017, (d) fall 2016 by sage‐grouse in 2018, and (e) fall 2015 by sage‐grouse in 2018. Gray bands represent 95% confidence intervals

Post‐fire treatments, including time since treatment, also influenced sage‐grouse behavior. Sage‐grouse were more likely to use areas with a greater proportion of herbicide treatment and of aerial seeding with forbs, and use was higher at earlier‐treated sites (Figure 4, Figure S5; Table 2c, Tables S12–S16). They also were more likely to use plots with a greater proportion of aerial seeding of sagebrush/grasses, a treatment conducted only in 2018 (Figure S6a; Table 2c, Table S15). In contrast, although sage‐grouse in 2017 and 2018 were more likely to use areas with a greater proportion of aerial seeding of sagebrush/forbs and of drill seeding, this use was lower at earlier‐treated sites (Figures S6b‐f and S7; Table 2c, Tables S12–S13, S15, S17–S19). Sage‐grouse also were less likely to use areas with steep slopes that were not targeted for drill seeding (Tables S15 and S17–S19).

## 4 DISCUSSION

Habitat selection by sage‐grouse after a large‐scale wildfire on the landscape was influenced by historical land cover and the recovery of vegetation after post‐fire restoration treatments. Selection patterns varied with time since treatment and by season, likely related to the timing of vegetation recovery on the landscape. Throughout the study, sage‐grouse strongly selected for recovering sagebrush and moderately selected for perennial bunchgrasses, immediately post‐fire they preferred forbs in summer, and in each year post‐fire they avoided exotic annual grasses. They also selected for areas treated with herbicide and with seeding of sagebrush, grasses, and forbs. These specific habitat preferences can have implications for prioritizing treatments in sage‐grouse habitat after catastrophic wildfires.

### 4.1 Post‐fire responses of sage‐grouse

Sage‐grouse responded to the increased presence of sagebrush on the landscape. The initial preference of sage‐grouse was for areas that had previously been dominated by low sagebrush cover. This selection may have been influenced by the presence of more low sagebrush than big sagebrush plants, presumably because low sagebrush occurred in rockier, lower‐fuel areas and were more likely to remain intact after the fire (C. Applestein, pers. obs.). However, by the third post‐fire year (2018), sage‐grouse began to shift their landscape use towards areas that historically were comprised of big sagebrush, perhaps responding to growth of this species.

Dietary preferences and forage quality of big and low sagebrush may partially explain these habitat selection patterns. Sage‐grouse are known to select low sagebrush plants because the leaves have more favorable secondary metabolites and fewer unfavorable metabolites and toxins than big sagebrush plants (Frye et al., 2013; Rosentreter, 2005). These differences may be especially relevant in winter when sage‐grouse rely on sagebrush for food (Patterson, 1952). Thus, post‐fire remnant patches of low sagebrush were likely important for winter foraging, as was found in central Oregon (Bruce et al., 2011). Although other factors, such as plant age, availability, or local adaptations may be relevant, sage‐grouse may prefer big sagebrush plants for cover but low sagebrush plants for nutrition (Frye et al., 2013), creating complex relationships between sage‐grouse and the two species of sagebrush found in our study area.

Sagebrush seedlings grew slowly over the three years post‐fire; thus, unsurprisingly, sage‐grouse responded to the highest sagebrush density bin in all seasons only in 2018. In the earliest year (2016), most areas occupied by sage‐grouse did not have any sagebrush plants. In later years, when some areas had higher densities of sagebrush plants, the birds disproportionately used these areas. By 2018, selection of high‐density sagebrush was particularly strong in winter, when sage‐grouse rely on sagebrush for food and cover. Sage‐grouse also did not respond positively to the sagebrush/forb seeding treatments until the second year (2017), but this also may have been because of the slow growth of sagebrush plants. In contrast, use of forb cover and forb seeding treatment areas was high in 2016, especially in summer, when little other food was available. However, this use generally decreased in later years. Further, across all years, sage‐grouse more strongly selected for the forb treatment conducted in winter 2016 than they did for the forb treatment conducted in the following year. The decreasing use of forbs in summer was surprising because sage‐grouse rely on summer habitats that provide succulent forbs for forage (Braun et al., 2005). In our study area, forb cover decreased over the three years as perennial bunchgrasses increased, which may partially explain the decreasing use of forbs over time.

Finally, sage‐grouse exhibited a strong negative selection against exotic annual grasses throughout the study. This response was consistent with selection for areas treated with imazapic, which is effective at reducing cover of exotic annual grasses (Applestein, Germino, & Fisk, 2018; Baker et al., 2009). This selection remained strong up to 2 years after treatment. This finding was expected because sage‐grouse are known to avoid habitats dominated by cheatgrass (Lockyer et al., 2015). In contrast, they positively responded to perennial bunchgrasses, including drill seeding treatments of grasses. Although sage‐grouse did not select drill seeded areas in 2016, their strongest responses to drill seeding appeared to be in the first year after treatment in 2017 and 2018. Additionally, the drill seeding in fall 2016 was applied in many of the same areas as the herbicide treatment in fall 2015. Delaying the seeding of grasses to 1 year after herbicide application can be effective in increasing cover of perennial bunchgrasses (Davies et al., 2014). In our study, mean cover of perennial bunchgrasses more than doubled between 2016 and 2017 in areas drill seeded in fall 2016. We suspect that sage‐grouse selected areas with grasses to provide cover from predators, given that sagebrush and other shrubs were lacking. Similarly, female sage‐grouse survival increased with higher herbaceous cover following a wildfire in Oregon (Foster et al., 2019). Thus, in our study area, integration of two treatment types, i.e. herbicide application and drill seeding of grasses, appeared to be influential in increasing post‐fire habitat use by sage‐grouse.

Our findings differ from a study analyzing the probability of the presence of sage‐grouse scat relative to post‐fire vegetation and treatments in our study area (Germino et al., *in press*). Similar to our study, the presence of scat was positively associated with sagebrush and negatively associated with exotic annual grass cover. However, post‐fire restoration treatments did not have any clear effects on the presence of scat. Thus, choice of monitoring approach, i.e. either capturing and tracking a limited number of animals for an extended time period or collecting scat from a greater number of animals at specific points in time, can affect inferences related to habitat selection.

### 4.2 Management and conservation implications for sage‐grouse

Despite decades of management and research efforts focused on sage‐grouse, habitat degradation due to wildfire has contributed to drastic population declines of sage‐grouse in much of their range (Coates et al., 2016; Connelly et al., 2000; Dudley et al., 2021). These trends appear to be applicable in our study area, where only six of the 10 leks that were active pre‐fire in 2015 remained active in 2020 (IDFG, unpublished data). Further, male attendance at leks in our study area declined 75% between 2015 and 2020, compared to a 47% decline in the larger sage‐grouse population. Thus, the Soda Wildfire appeared to be a contributing factor to local short‐term declines in sage‐grouse numbers.

Quantitative evidence of three decades of population, wildfire, and climate data has recently linked long‐term declines in sage‐grouse populations to chronic effects of wildfire (Coates et al., 2016). Fires in the western portion of sage‐grouse range during this time period have substantially increased in frequency and size (Brooks et al., 2015). Further, if current wildfire trends continue, model projections suggest that, in the next three decades, sage‐grouse populations will be reduced to <50% of their current levels (Coates et al., 2016).

The positive feedback loop between cheatgrass invasion and wildfire is thought to be the primary mechanism responsible for the ecosystem‐level transformation of the sagebrush steppe in the Intermountain West (Chambers et al., 2014; Coates et al., 2016). Cheatgrass is a fine fuel that is more likely to burn than other land cover types, easily re‐establishes after fire, outcompetes native plants, and contributes to the continued loss of sagebrush habitat (Boyte et al., 2016; Bradley et al., 2018). Further, Wyoming Big Sagebrush ecosystems, such as that in our study area, are the least resistant of all sagebrush ecosystems to cheatgrass invasion and dominance (Chambers et al., 2014). Thus, suppression efforts that substantially reduce the rate of wildfires in sagebrush ecosystems, especially those ecosystems where resistance to cheatgrass is low, could be critical to slow declines in, or stabilize or increase, populations of sage‐grouse (Coates et al., 2016; Foster et al., 2019).

## 5 CONCLUSIONS

Although sagebrush seedlings grew slowly over the three years of our study, sage‐grouse showed a strong positive response to their presence, including in areas treated to improve density and numbers of sagebrush plants. Conversely, sage‐grouse avoided exotic annual grasses and preferred areas that had been treated with herbicide then re‐seeded with perennial grasses. Previous work has shown that wildfire prevention and suppression practices can better insulate sage‐grouse populations from the cheatgrass‐fire cycle than can post‐fire restoration. However, when suppression efforts fail and wildfires do occur, our study shows that post‐fire restoration treatments, especially cheatgrass control, can contribute to the recovery of sage‐grouse habitat. Although active post‐fire treatments are understood to be necessary to assist in the recovery of sagebrush landscapes, our study is one of the first to describe how these treatments influence sage‐grouse activity and habitat selection in the years immediately following a wildfire. Sage‐grouse locations were highly concentrated in areas that received some of the most intense treatment efforts. Thus, in the event of a wildfire, post‐fire restoration treatments will likely be important in the recovery of sage‐grouse populations and their habitat.

## CONFLICT OF INTEREST

The authors declare no conflict of interest.

## AUTHOR CONTRIBUTIONS

**Sharon A. Poessel:** Conceptualization (equal); Data curation (equal); Formal analysis (lead); Methodology (equal); Visualization (equal); Writing – original draft (lead); Writing – review & editing (equal). **David M. Barnard:** Data curation (equal); Investigation (equal); Methodology (equal); Writing – review & editing (equal). **Cara Applestein:** Data curation (equal); Investigation (equal); Methodology (equal); Writing – review & editing (equal). **Matthew J. Germino:** Conceptualization (equal); Data curation (equal); Funding acquisition (equal); Investigation (equal); Methodology (equal); Supervision (equal); Writing – review & editing (equal). **Ethan A. Ellsworth:** Conceptualization (equal); Data curation (equal); Funding acquisition (equal); Investigation (equal); Methodology (equal); Resources (equal); Writing – review & editing (equal). **Don Major:** Conceptualization (equal); Data curation (equal); Investigation (equal); Methodology (equal); Resources (equal); Writing – review & editing (equal). **Ann Moser:** Data curation (equal); Investigation (equal); Methodology (equal); Resources (equal); Writing – review & editing (equal). **Todd E. Katzner:** Conceptualization (equal); Data curation (equal); Methodology (equal); Supervision (equal); Visualization (equal); Writing – original draft (supporting); Writing – review & editing (equal).

## Supporting information

Appendix S1

## Data Availability

Data used in habitat selection analyses are available in the ScienceBase digital repository: https://doi.org/10.5066/P9RH792J.
