# Supplementary material for: Greater sage‐grouse respond positively to intensive post‐fire restoration treatments
Source: Ecol Evol. 2022 Mar 21;12(3):e8671. doi: 10.1002/ece3.8671 (PMC8938311; doi:10.1002/ece3.8671)
Supplement: Supplementary file 1 — Appendix S1 [file ECE3-12-e8671-s001.docx]

**Greater sage-grouse respond positively to
intensive post-fire restoration treatments**

**Supporting Information**

**Appendix S1**

**Additional Methods**

To account for responses to the correlated variables that we excluded from previous analysis, we ran additional models to evaluate sage-grouse responses to pre-fire land cover, post-fire vegetation, and post-fire treatments. To evaluate sage-grouse response to pre-fire land cover, we ran one additional model for each year in which the only fixed effect was the interaction term between season and the sagebrush variable excluded from the primary models described in the main text (i.e., low sagebrush in 2016 and 2017 and big sagebrush in 2018). This resulted in three additional models, one for each year, evaluating sage-grouse response to pre-fire land cover.

To evaluate sage-grouse response to previously excluded post-fire vegetation predictors, we ran one additional model for each year in which the only fixed effects were interaction terms between season and each of sagebrush density bins 2 (1-100 plants/ha), 3 (101-1,000 plants/ha), and 4 (>1,000 plants/ha); we did not consider in our models density bin 1 (0 plants/ha) because it was negatively correlated with bins 3 and 4. Because the use and available buffers were primarily composed of density bin 1 in 2016, our analyses generated large parameter coefficients and models did not converge. Consequently, we did not interpret the 2016 model for sagebrush density. This resulted in two additional models evaluating sage-grouse response to post-fire vegetation.

To evaluate sage-grouse response to previously excluded post-fire treatment predictors, we ran one additional model for 2016 and two for each of 2017 and 2018. In the additional model for 2016, the three fixed effects were season, drill seeding conducted in fall 2015, and slope. In the first additional model for 2017, the four fixed effects were season, drill seeding conducted in fall 2015 and fall 2016, and slope. The second additional model for 2017 included two fixed effects, one for season and one for herbicide application in fall 2015. Finally, in the first additional model for 2018, the four fixed effects were season, drill seeding conducted in fall 2016 and fall 2017, and slope. The second additional model for 2018 included three fixed effects, one for season and two for herbicide application in both fall 2015 and fall 2016. This resulted in five additional models evaluating sage-grouse response to post-fire treatments.

**
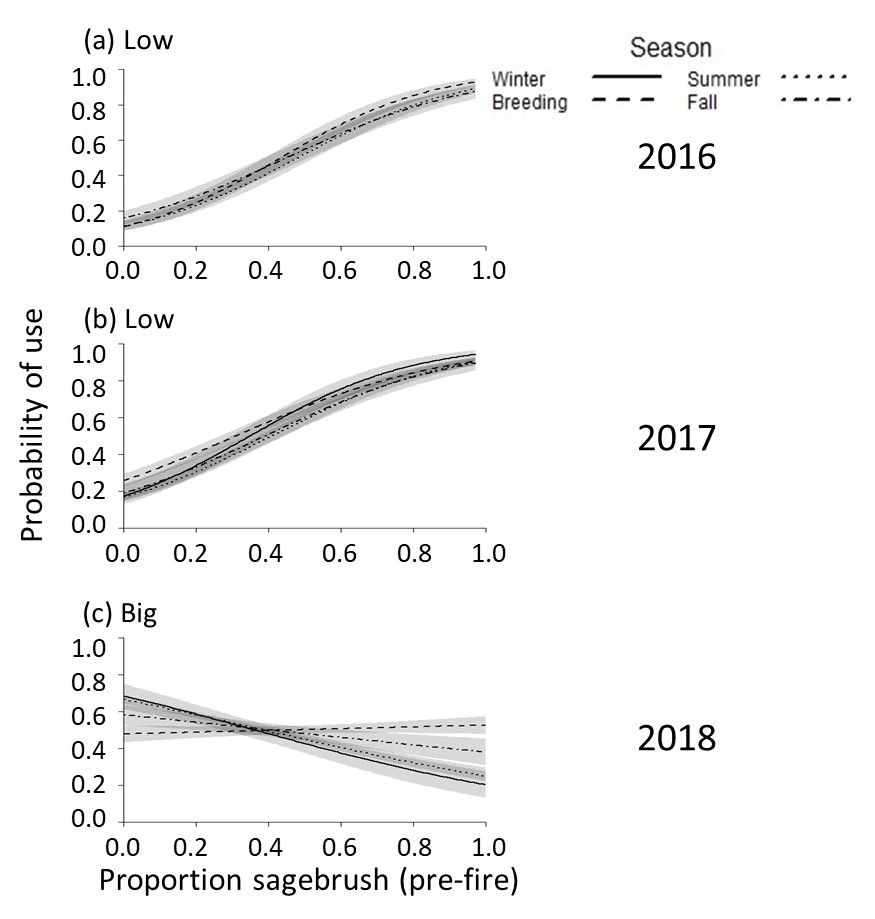
**

**FIGURE S1** Probability of use by greater sage-grouse, by season, year (2016-2018), and proportion of 500-m buffers around use and available points that were composed of pre-fire land cover types, in the Soda Wildfire study area. Plots show response to pre-fire low sagebrush cover by sage-grouse in (a) 2016 and (b) 2017, and to pre-fire big sagebrush cover by sage-grouse in (c) 2018. Winter season was not represented in 2016. Gray bands represent 95% confidence intervals.


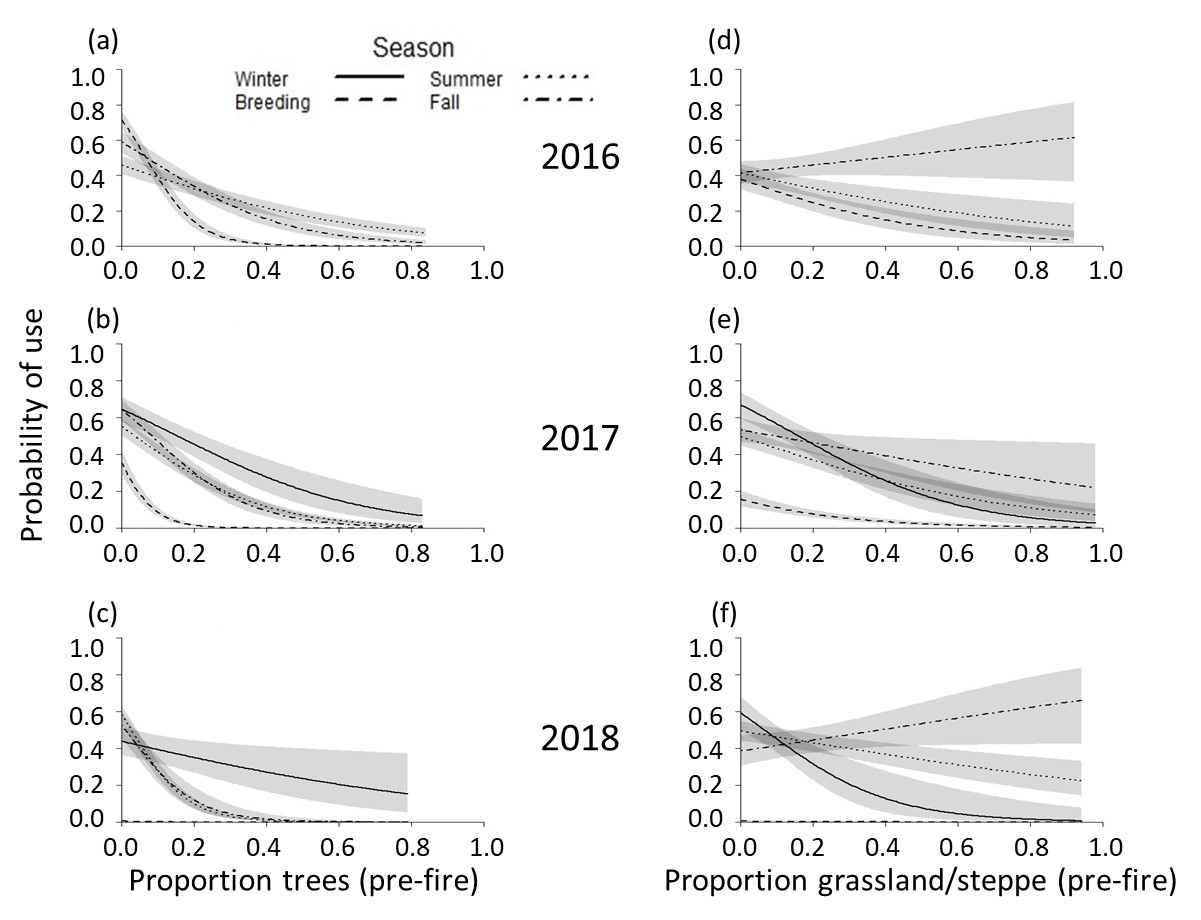


**FIGURE S2** Probability of use by greater sage-grouse, by season, year (2016-2018), and proportion of 500-m buffers around use and available points that were composed of pre-fire land cover types, in the Soda Wildfire study area. Plots show response to pre-fire trees by sage-grouse in (a) 2016, (b) 2017, and (c) 2018, and to pre-fire grassland and steppe by sage-grouse in (d) 2016, (e) 2017, and (f) 2018. Winter season was not represented in 2016. Gray bands represent 95% confidence intervals.


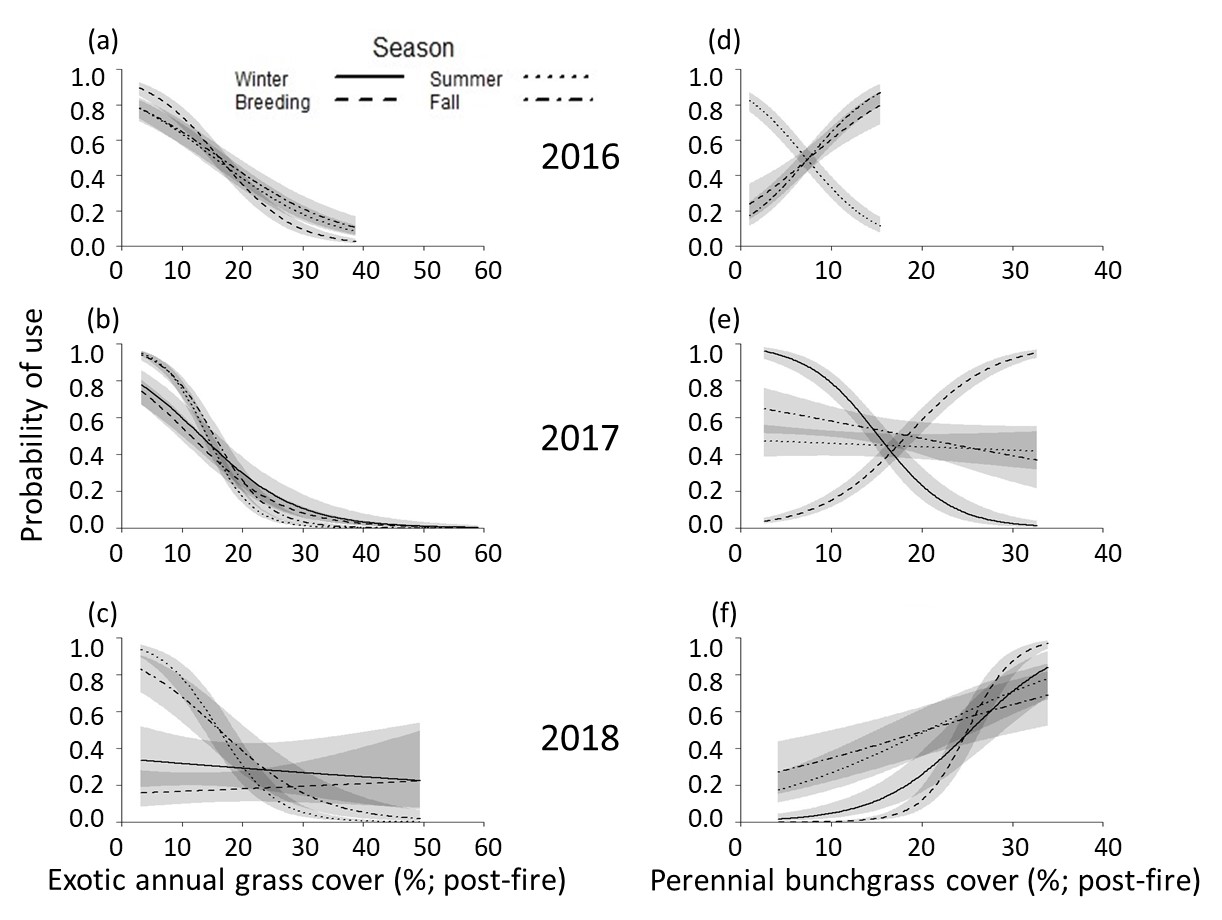


**FIGURE S3** Probability of use by greater sage-grouse, by season, year (2016-2018), and mean percent cover of post-fire vegetation types in 500-m buffers around use and available points, in the Soda Wildfire study area. Plots show response by sage-grouse to post-fire exotic annual grass cover in (a) 2016, (b) 2017, and (c) 2018, and to post-fire perennial bunchgrass cover in (d) 2016, (e) 2017, and (f) 2018. Winter season was not represented in 2016. Gray bands represent 95% confidence intervals.

**
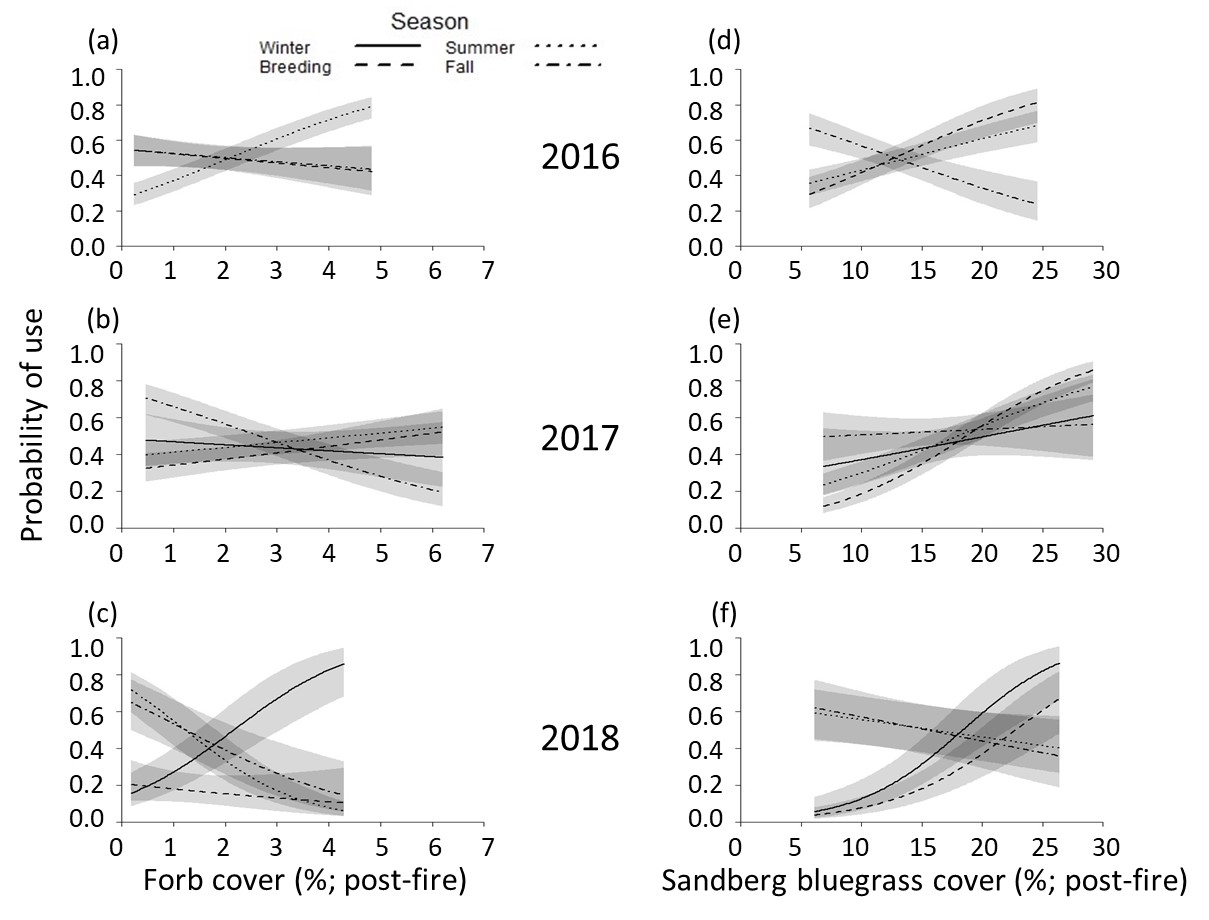
**

**FIGURE S4** Probability of use by greater sage-grouse, by season, year (2016-2018), and mean percent cover of post-fire vegetation types in 500-m buffers around use and available points, in the Soda Wildfire study area. Plots show response by sage-grouse to post-fire forb cover in (a) 2016, (b) 2017, and (c) 2018, and to post-fire Sandberg bluegrass cover in (d) 2016, (e) 2017, and (f) 2018. Winter season was not represented in 2016. Gray bands represent 95% confidence intervals.


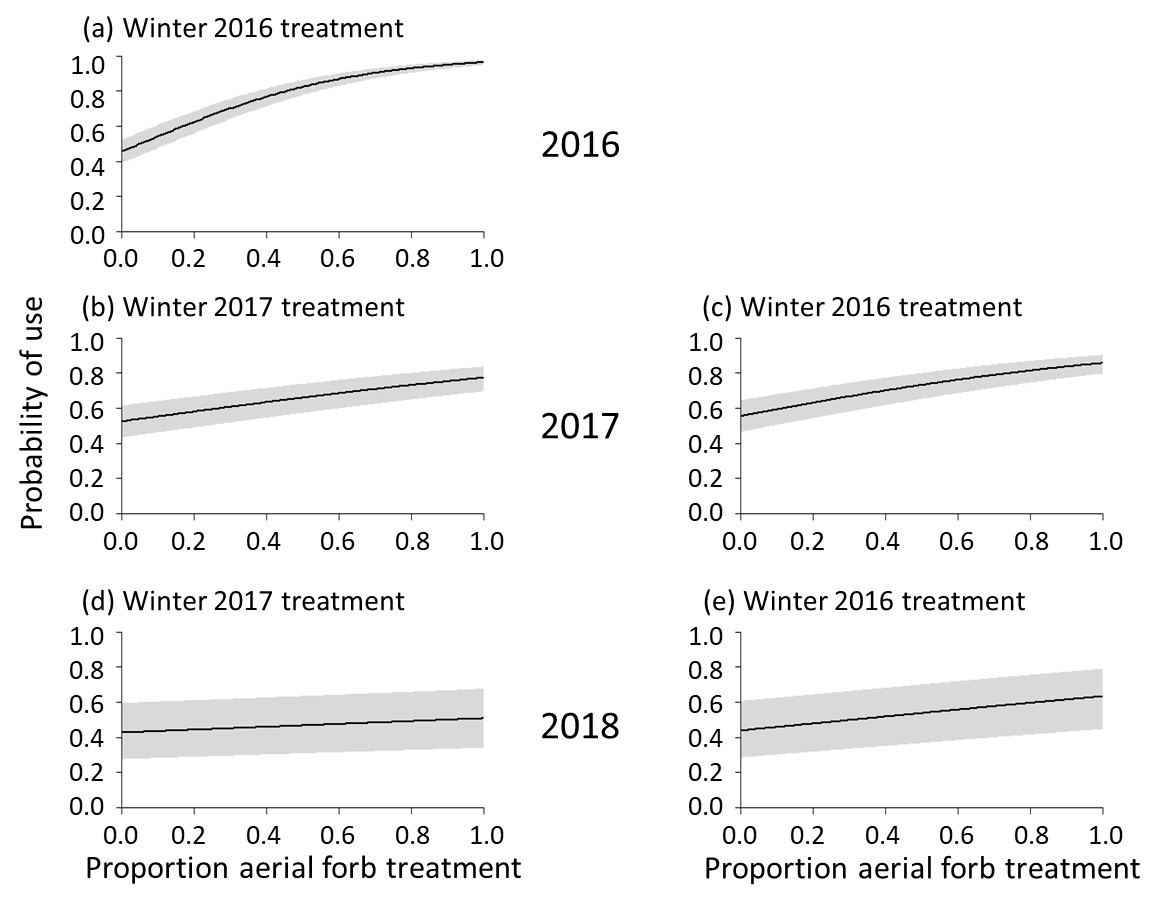


**FIGURE S5** Probability of use by greater sage-grouse, by year (2016-2018) and proportion of 500-m buffers around use and available points that were composed of post-fire treatment areas, in the Soda Wildfire study area. Plots show response to treatment with aerial seeding of forbs in (a) winter 2016 by sage-grouse in 2016, (b) winter 2017 by sage-grouse in 2017, (c) winter 2016 by sage-grouse in 2017, (d) winter 2017 by sage-grouse in 2018, and (e) winter 2016 by sage-grouse in 2018. Gray bands represent 95% confidence intervals.


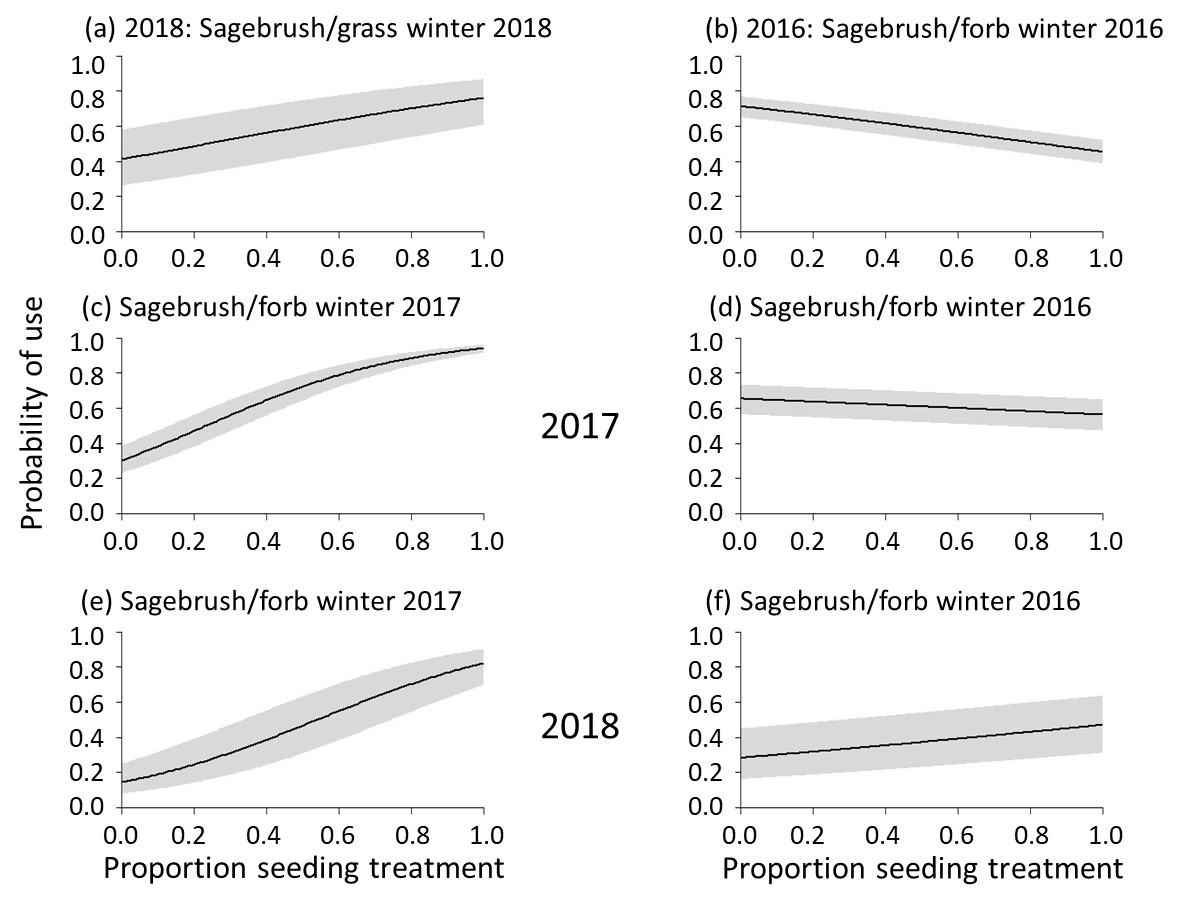


**FIGURE S6** Probability of use by greater sage-grouse, by year (2016-2018) and proportion of 500-m buffers around use and available points that were composed of post-fire treatment areas, in the Soda Wildfire study area. Plots show response to treatment with aerial seeding of a sagebrush/grass mix in (a) winter 2018 by sage-grouse in 2018, and a sagebrush/forb mix in (b) winter 2016 by sage-grouse in 2016, (c) winter 2017 by sage-grouse in 2017, (d) winter 2016 by sage-grouse in 2017, (e) winter 2017 by sage-grouse in 2018, and (f) winter 2016 by sage-grouse in 2018. Gray bands represent 95% confidence intervals.


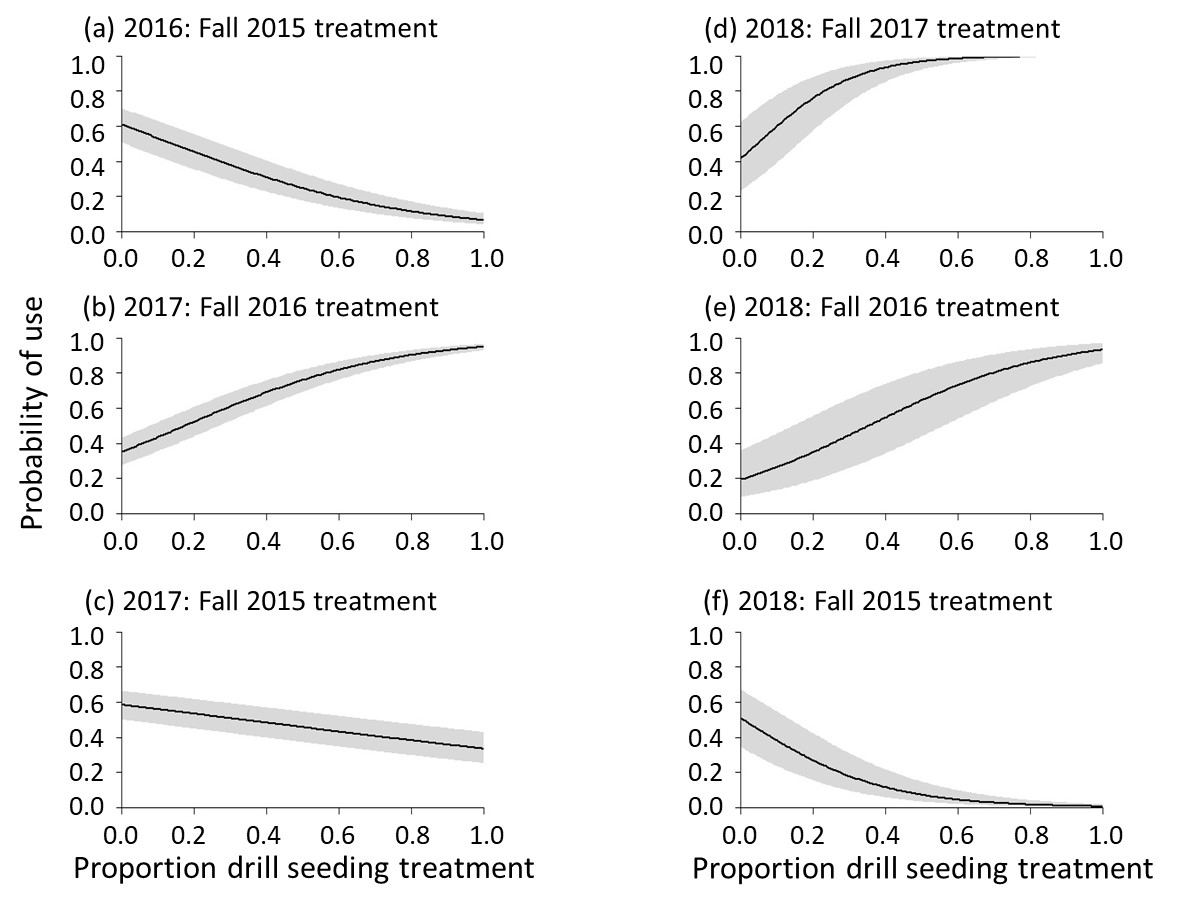


**FIGURE S7** Probability of use by greater sage-grouse, by year (2016-2018) and proportion of 500-m buffers around use and available points that were composed of post-fire treatment areas, in the Soda Wildfire study area. Plots show response to treatment with drill seeding in (a) fall 2015 by sage-grouse in 2016, (b) fall 2016 by sage-grouse in 2017, (c) fall 2015 by sage-grouse in 2017, (d) fall 2017 by sage-grouse in 2018, (e) fall 2016 by sage-grouse in 2018, and (f) fall 2015 by sage-grouse in 2018. Gray bands represent 95% confidence intervals.

**TABLE S1** Model-averaged coefficients, adjusted *SE*s, *z*-values, and 95% confidence intervals (CI) from the four best-performing linear mixed effects models (model weights ≥0.01) explaining selection of pre-fire land cover (except low sagebrush) by greater sage-grouse in the Soda Wildfire area in southern Idaho and Oregon during 2016. Reference variable for season was breeding; sage-grouse were not tracked in winter of this year. Variables with CI ranges that did not overlap zero are important variables.

| **Variable** | **Coefficient** | **Adjusted *SE*** | ***z*-value** | **Lower CI** | **Upper CI** |
| --- | --- | --- | --- | --- | --- |
| Intercept | -0.79 | 0.12 | 6.45 | -1.03 | -0.55 |
| Season-summer | -0.21 | 0.11 | 1.94 | -0.41 | 0.00 |
| Season-fall | 0.23 | 0.12 | 1.90 | -0.01 | 0.47 |
| Big sagebrush | -2.35 | 0.17 | 13.76 | -2.68 | -2.01 |
| Introduced grassland | -4.71 | 0.59 | 8.02 | -5.87 | -3.56 |
| Grassland and steppe | -0.67 | 0.13 | 4.98 | -0.93 | -0.40 |
| Trees | -4.03 | 0.27 | 15.00 | -4.56 | -3.51 |
| Summer*big sagebrush | -0.35 | 0.24 | 1.46 | -0.81 | 0.12 |
| Fall*big sagebrush | 0.06 | 0.21 | 0.26 | -0.36 | 0.47 |
| Summer*introduced grassland | 0.16 | 0.57 | 0.27 | -0.96 | 1.27 |
| Fall*introduced grassland | 0.23 | 0.65 | 0.35 | -1.05 | 1.50 |
| Summer*grassland and steppe | 0.26 | 0.17 | 1.52 | -0.08 | 0.60 |
| Fall*grassland and steppe | 0.85 | 0.19 | 4.46 | 0.47 | 1.22 |
| Summer*trees | 3.22 | 0.28 | 11.58 | 2.67 | 3.76 |
| Fall*trees | 2.51 | 0.29 | 8.60 | 1.94 | 3.08 |

**TABLE S2** Coefficients, *SE*s, *z*-values, and 95% confidence intervals (CI) from the best-performing linear mixed effects model (model weight 0.99) explaining selection of pre-fire land cover (except low sagebrush) by greater sage-grouse in the Soda Wildfire area in southern Idaho and Oregon during 2017. Reference variable for season was winter. Variables with CI ranges that did not overlap zero are important variables.

| **Variable** | **Coefficient** | ***SE*** | ***z*-value** | **Lower CI** | **Upper CI** |
| --- | --- | --- | --- | --- | --- |
| Intercept | 0.29 | 0.13 | 2.23 | 0.03 | 0.55 |
| Season-breeding | -2.43 | 0.16 | -14.96 | -2.75 | -2.11 |
| Season-summer | -0.55 | 0.11 | -5.04 | -0.76 | -0.33 |
| Season-fall | -0.26 | 0.12 | -2.17 | -0.50 | -0.03 |
| Big sagebrush | -2.19 | 0.22 | -9.98 | -2.62 | -1.76 |
| Introduced grassland | -0.80 | 0.17 | -4.80 | -1.12 | -0.47 |
| Grassland and steppe | -0.94 | 0.18 | -5.20 | -1.29 | -0.58 |
| Trees | -1.14 | 0.20 | -5.73 | -1.53 | -0.75 |
| Breeding*big sagebrush | 1.49 | 0.24 | 6.15 | 1.01 | 1.96 |
| Summer*big sagebrush | 0.20 | 0.23 | 0.84 | -0.26 | 0.66 |
| Fall*big sagebrush | 0.00 | 0.29 | -0.01 | -0.57 | 0.56 |
| Breeding*introduced grassland | -14.58 | 0.88 | -16.53 | -16.31 | -12.85 |
| Summer*introduced grassland | -2.60 | 0.37 | -6.93 | -3.33 | -1.86 |
| Fall*introduced grassland | -0.83 | 0.34 | -2.46 | -1.49 | -0.17 |
| Breeding*grassland and steppe | 0.05 | 0.21 | 0.25 | -0.36 | 0.47 |
| Summer*grassland and steppe | 0.38 | 0.20 | 1.93 | -0.01 | 0.77 |
| Fall*grassland and steppe | 0.63 | 0.23 | 2.80 | 0.19 | 1.08 |
| Breeding*trees | -4.06 | 0.35 | -11.58 | -4.75 | -3.38 |
| Summer*trees | -0.49 | 0.21 | -2.34 | -0.89 | -0.08 |
| Fall*trees | -0.96 | 0.26 | -3.65 | -1.48 | -0.45 |

**TABLE S3** Coefficients, *SE*s, *z*-values, and 95% confidence intervals (CI) from the best-performing linear mixed effects model (model weight 1.00) explaining selection of pre-fire land cover (except big sagebrush) by greater sage-grouse in the Soda Wildfire area in southern Idaho and Oregon during 2018. Reference variable for season was winter. Variables with CI ranges that did not overlap zero are important variables.

| **Variable** | **Coefficient** | ***SE*** | ***z*-value** | **Lower CI** | **Upper CI** |
| --- | --- | --- | --- | --- | --- |
| Intercept | -0.23 | 0.15 | -1.51 | -0.52 | 0.07 |
| Season-breeding | -4.05 | 0.32 | -12.85 | -4.67 | -3.43 |
| Season-summer | -0.31 | 0.14 | -2.26 | -0.57 | -0.04 |
| Season-fall | -0.31 | 0.18 | -1.78 | -0.66 | 0.03 |
| Low sagebrush | 1.34 | 0.23 | 5.72 | 0.88 | 1.80 |
| Introduced grassland | -0.05 | 0.22 | -0.21 | -0.48 | 0.39 |
| Grassland and steppe | -1.22 | 0.32 | -3.79 | -1.85 | -0.59 |
| Trees | -0.54 | 0.23 | -2.35 | -1.00 | -0.09 |
| Breeding*low sagebrush | -1.61 | 0.28 | -5.76 | -2.15 | -1.06 |
| Summer*low sagebrush | 0.47 | 0.24 | 1.95 | 0.00 | 0.95 |
| Fall*low sagebrush | -0.37 | 0.29 | -1.26 | -0.93 | 0.20 |
| Breeding*introduced grassland | -32.97 | 2.11 | -15.66 | -37.10 | -28.85 |
| Summer*introduced grassland | -0.91 | 0.26 | -3.49 | -1.43 | -0.40 |
| Fall*introduced grassland | -3.12 | 0.68 | -4.62 | -4.44 | -1.80 |
| Breeding*grassland and steppe | 0.51 | 0.35 | 1.47 | -0.17 | 1.19 |
| Summer*grassland and steppe | 0.94 | 0.33 | 2.87 | 0.30 | 1.59 |
| Fall*grassland and steppe | 1.47 | 0.35 | 4.24 | 0.79 | 2.15 |
| Breeding*trees | -2.69 | 0.40 | -6.80 | -3.47 | -1.91 |
| Summer*trees | -3.18 | 0.30 | -10.61 | -3.77 | -2.59 |
| Fall*trees | -2.55 | 0.48 | -5.33 | -3.49 | -1.62 |

**TABLE S4** Coefficients, *SE*s, *z*-values, and 95% confidence intervals (CI) from the best-performing linear mixed effects model (model weight 0.96) explaining selection of pre-fire low sagebrush by greater sage-grouse in the Soda Wildfire area in southern Idaho and Oregon during 2016. Reference variable for season was breeding; sage-grouse were not tracked in winter of this year. Variables with CI ranges that did not overlap zero are important variables.

| **Variable** | **Coefficient** | ***SE*** | ***z*-value** | **Lower CI** | **Upper CI** |
| --- | --- | --- | --- | --- | --- |
| Intercept | -0.19 | 0.11 | -1.80 | -0.41 | 0.02 |
| Season-summer | -0.18 | 0.07 | -2.50 | -0.33 | -0.04 |
| Season-fall | -0.02 | 0.09 | -0.19 | -0.19 | 0.16 |
| Low sagebrush | 2.60 | 0.14 | 18.86 | 2.33 | 2.87 |
| Summer*low sagebrush | -0.26 | 0.16 | -1.65 | -0.57 | 0.05 |
| Fall*low sagebrush | -0.57 | 0.18 | -3.18 | -0.92 | -0.22 |

**TABLE S5** Model-averaged coefficients, adjusted *SE*s, *z*-values, and 95% confidence intervals (CI) from the two best-performing linear mixed effects models (model weights ≥0.01) explaining selection of pre-fire low sagebrush by greater sage-grouse in the Soda wildfire area in southern Idaho and Oregon during 2017. Reference variable for season was winter. Variables with CI ranges that did not overlap zero are important variables.

| **Variable** | **Coefficient** | **Adjusted *SE*** | ***z*-value** | **Lower CI** | **Upper CI** |
| --- | --- | --- | --- | --- | --- |
| Intercept | 0.19 | 0.12 | 1.67 | -0.03 | 0.42 |
| Season-breeding | 0.09 | 0.10 | 0.93 | -0.10 | 0.29 |
| Season-summer | -0.26 | 0.10 | 2.59 | -0.45 | -0.06 |
| Season-fall | -0.19 | 0.11 | 1.63 | -0.41 | 0.04 |
| Low sagebrush | 2.40 | 0.22 | 11.04 | 1.97 | 2.82 |
| Breeding*low sagebrush | -0.53 | 0.26 | 2.06 | -1.04 | -0.02 |
| Summer*low sagebrush | -0.27 | 0.22 | 1.24 | -0.70 | 0.16 |
| Fall*low sagebrush | -0.39 | 0.27 | 1.43 | -0.91 | 0.14 |

**TABLE S6** Coefficients, *SE*s, *z*-values, and 95% confidence intervals (CI) from the best-performing linear mixed effects model (model weight 1.00) explaining selection of pre-fire big sagebrush by greater sage-grouse in the Soda Wildfire area in southern Idaho and Oregon during 2018. Reference variable for season was winter. Variables with CI ranges that did not overlap zero are important variables.

| **Variable** | **Coefficient** | ***SE*** | ***z*-value** | **Lower CI** | **Upper CI** |
| --- | --- | --- | --- | --- | --- |
| Intercept | -0.02 | 0.09 | -0.17 | -0.20 | 0.17 |
| Season-breeding | 0.00 | 0.10 | -0.03 | -0.20 | 0.20 |
| Season-summer | 0.04 | 0.10 | 0.43 | -0.15 | 0.23 |
| Season-fall | 0.04 | 0.12 | 0.35 | -0.19 | 0.27 |
| Big sagebrush | -1.08 | 0.19 | -5.78 | -1.44 | -0.71 |
| Breeding*big sagebrush | 1.17 | 0.20 | 5.75 | 0.77 | 1.57 |
| Summer*big sagebrush | 0.18 | 0.19 | 0.93 | -0.20 | 0.56 |
| Fall*big sagebrush | 0.66 | 0.22 | 3.02 | 0.23 | 1.09 |

**TABLE S7** Coefficients, *SE*s, *z*-values, and 95% confidence intervals (CI) from the best-performing linear mixed effects model (model weight 1.00) explaining selection of post-fire vegetation (except sagebrush density) by greater sage-grouse in the Soda Wildfire area in southern Idaho and Oregon during 2016. Reference variable for season was breeding; sage-grouse were not tracked in winter of this year. Variables with CI ranges that did not overlap zero are important variables.

| **Variable** | **Coefficient** | ***SE*** | ***z*-value** | **Lower CI** | **Upper CI** |
| --- | --- | --- | --- | --- | --- |
| Intercept | 1.73 | 0.29 | 5.89 | 1.15 | 2.31 |
| Season-summer | -3.57 | 0.32 | -10.99 | -4.21 | -2.93 |
| Season-fall | 0.02 | 0.37 | 0.04 | -0.70 | 0.73 |
| Exotic annual grass cover | -2.28 | 0.15 | -15.23 | -2.57 | -1.98 |
| Forb cover | -0.22 | 0.18 | -1.25 | -0.57 | 0.13 |
| Perennial bunchgrass cover | 2.57 | 0.52 | 4.96 | 1.55 | 3.58 |
| Sandberg bluegrass cover | 0.83 | 0.16 | 5.04 | 0.50 | 1.15 |
| Summer*exotic annual grass cover | 0.84 | 0.17 | 5.00 | 0.51 | 1.17 |
| Fall*exotic annual grass cover | 0.94 | 0.21 | 4.52 | 0.53 | 1.35 |
| Summer*forb cover | 1.24 | 0.20 | 6.22 | 0.85 | 1.62 |
| Fall*forb cover | 0.03 | 0.24 | 0.11 | -0.44 | 0.49 |
| Summer*perennial bunchgrass cover | -6.22 | 0.60 | -10.30 | -7.40 | -5.03 |
| Fall*perennial bunchgrass cover | 0.93 | 0.68 | 1.38 | -0.39 | 2.26 |
| Summer*Sandberg bluegrass cover | -0.35 | 0.18 | -1.89 | -0.71 | 0.01 |
| Fall*Sandberg bluegrass cover | -1.48 | 0.22 | -6.66 | -1.92 | -1.05 |

**TABLE S8** Coefficients, *SE*s, *z*-values, and 95% confidence intervals (CI) from the best-performing linear mixed effects model (model weight 1.00) explaining selection of post-fire vegetation (except sagebrush density) by greater sage-grouse in the Soda Wildfire area in southern Idaho and Oregon during 2017. Reference variable for season was winter. Variables with CI ranges that did not overlap zero are important variables.

| **Variable** | **Coefficient** | ***SE*** | ***z*-value** | **Lower CI** | **Upper CI** |
| --- | --- | --- | --- | --- | --- |
| Intercept | -0.15 | 0.22 | -0.69 | -0.58 | 0.27 |
| Season-breeding | -0.83 | 0.19 | -4.39 | -1.20 | -0.46 |
| Season-summer | -0.42 | 0.18 | -2.31 | -0.77 | -0.06 |
| Season-fall | 0.32 | 0.20 | 1.60 | -0.07 | 0.71 |
| Exotic annual grass cover | -1.75 | 0.24 | -7.21 | -2.23 | -1.28 |
| Forb cover | -0.14 | 0.21 | -0.65 | -0.56 | 0.28 |
| Perennial bunchgrass cover | -3.66 | 0.45 | -8.20 | -4.54 | -2.78 |
| Sandberg bluegrass cover | 0.34 | 0.26 | 1.31 | -0.17 | 0.84 |
| Breeding*exotic annual grass cover | -0.06 | 0.28 | -0.20 | -0.60 | 0.49 |
| Summer*exotic annual grass cover | -2.01 | 0.27 | -7.45 | -2.54 | -1.48 |
| Fall*exotic annual grass cover | -1.42 | 0.34 | -4.24 | -2.08 | -0.76 |
| Breeding*forb cover | 0.44 | 0.25 | 1.78 | -0.05 | 0.92 |
| Summer*forb cover | 0.36 | 0.23 | 1.59 | -0.08 | 0.80 |
| Fall*forb cover | -0.70 | 0.26 | -2.73 | -1.20 | -0.20 |
| Breeding* perennial bunchgrass cover | 6.69 | 0.48 | 13.84 | 5.74 | 7.64 |
| Summer* perennial bunchgrass cover | 3.55 | 0.47 | 7.59 | 2.63 | 4.47 |
| Fall* perennial bunchgrass cover | 3.10 | 0.53 | 5.87 | 2.07 | 4.14 |
| Breeding* Sandberg bluegrass cover | 0.79 | 0.28 | 2.85 | 0.25 | 1.34 |
| Summer* Sandberg bluegrass cover | 0.37 | 0.27 | 1.38 | -0.16 | 0.90 |
| Fall* Sandberg bluegrass cover | -0.26 | 0.31 | -0.84 | -0.86 | 0.34 |

**TABLE S9** Coefficients, *SE*s, *z*-values, and 95% confidence intervals (CI) from the best-performing linear mixed effects model (model weight 1.00) explaining selection of post-fire vegetation (except sagebrush density) by greater sage-grouse in the Soda Wildfire area in southern Idaho and Oregon during 2018. Reference variable for season was winter. Variables with CI ranges that did not overlap zero are important variables.

| **Variable** | **Coefficient** | ***SE*** | ***z*-value** | **Lower CI** | **Upper CI** |
| --- | --- | --- | --- | --- | --- |
| Intercept | -1.34 | 0.31 | -4.26 | -1.96 | -0.72 |
| Season-breeding | -2.60 | 0.22 | -11.74 | -3.04 | -2.17 |
| Season-summer | 0.27 | 0.19 | 1.45 | -0.09 | 0.63 |
| Season-fall | 0.65 | 0.23 | 2.82 | 0.20 | 1.11 |
| Exotic annual grass cover | -0.17 | 0.27 | -0.61 | -0.70 | 0.37 |
| Forb cover | 1.78 | 0.32 | 5.57 | 1.15 | 2.40 |
| Perennial bunchgrass cover | 2.84 | 0.40 | 7.10 | 2.06 | 3.62 |
| Sandberg bluegrass cover | 1.53 | 0.30 | 5.07 | 0.94 | 2.12 |
| Breeding*exotic annual grass cover | 0.30 | 0.36 | 0.81 | -0.42 | 1.01 |
| Summer*exotic annual grass cover | -2.75 | 0.31 | -8.93 | -3.36 | -2.15 |
| Fall*exotic annual grass cover | -1.52 | 0.37 | -4.08 | -2.25 | -0.79 |
| Breeding*forb cover | -2.18 | 0.51 | -4.30 | -3.17 | -1.19 |
| Summer*forb cover | -3.64 | 0.35 | -10.48 | -4.32 | -2.96 |
| Fall*forb cover | -2.99 | 0.45 | -6.72 | -3.87 | -2.12 |
| Breeding* perennial bunchgrass cover | 2.89 | 0.51 | 5.66 | 1.89 | 3.89 |
| Summer* perennial bunchgrass cover | -1.46 | 0.41 | -3.56 | -2.26 | -0.66 |
| Fall* perennial bunchgrass cover | -1.97 | 0.46 | -4.25 | -2.87 | -1.06 |
| Breeding* Sandberg bluegrass cover | -0.23 | 0.35 | -0.67 | -0.92 | 0.45 |
| Summer* Sandberg bluegrass cover | -1.78 | 0.31 | -5.68 | -2.39 | -1.16 |
| Fall* Sandberg bluegrass cover | -1.88 | 0.36 | -5.24 | -2.58 | -1.17 |

**TABLE S10** Coefficients, *SE*s, *z*-values, and 95% confidence intervals (CI) from the best-performing linear mixed effects model (model weight 1.00) explaining selection of post-fire sagebrush density by greater sage-grouse in the Soda Wildfire area in southern Idaho and Oregon during 2017. Reference variable for season was winter. Bin 2 was 1-100 sagebrush plants/ha, bin 3 was 101-1,000 sagebrush plants/ha, and bin 4 was >1,000 sagebrush plants/ha. Variables with CI ranges that did not overlap zero are important variables.

| **Variable** | **Coefficient** | ***SE*** | ***z*-value** | **Lower CI** | **Upper CI** |
| --- | --- | --- | --- | --- | --- |
| Intercept | 0.03 | 0.23 | 0.15 | -0.41 | 0.48 |
| Season-breeding | 0.07 | 0.14 | 0.48 | -0.21 | 0.34 |
| Season-summer | 0.35 | 0.14 | 2.55 | 0.08 | 0.62 |
| Season-fall | 0.46 | 0.15 | 3.03 | 0.16 | 0.75 |
| Sagebrush density bin 2 | 0.22 | 0.10 | 2.18 | 0.02 | 0.42 |
| Sagebrush density bin 3 | 3.18 | 0.29 | 11.10 | 2.62 | 3.74 |
| Sagebrush density bin 4 | -2.94 | 0.57 | -5.18 | -4.06 | -1.83 |
| Breeding*sagebrush density bin 2 | -1.13 | 0.16 | -7.13 | -1.44 | -0.82 |
| Summer*sagebrush density bin 2 | -0.37 | 0.11 | -3.36 | -0.59 | -0.16 |
| Fall*sagebrush density bin 2 | -0.25 | 0.13 | -1.95 | -0.50 | 0.00 |
| Breeding*sagebrush density bin 3 | -2.52 | 0.31 | -8.06 | -3.14 | -1.91 |
| Summer*sagebrush density bin 3 | -1.48 | 0.30 | -4.96 | -2.06 | -0.89 |
| Fall*sagebrush density bin 3 | -1.64 | 0.35 | -4.63 | -2.34 | -0.95 |
| Breeding*sagebrush density bin 4 | 5.84 | 0.59 | 9.85 | 4.67 | 7.00 |
| Summer*sagebrush density bin 4 | 4.43 | 0.58 | 7.66 | 3.29 | 5.56 |
| Fall*sagebrush density bin 4 | 4.32 | 0.61 | 7.06 | 3.12 | 5.52 |

**TABLE S11** Coefficients, *SE*s, *z*-values, and 95% confidence intervals (CI) from the best-performing linear mixed effects model (model weight 1.00) explaining selection of post-fire sagebrush density by greater sage-grouse in the Soda Wildfire area in southern Idaho and Oregon during 2018. Reference variable for season was winter. Bin 2 was 1-100 sagebrush plants/ha, bin 3 was 101-1,000 sagebrush plants/ha, and bin 4 was >1,000 sagebrush plants/ha. Variables with CI ranges that did not overlap zero are important variables.

| **Variable** | **Coefficient** | ***SE*** | ***z*-value** | **Lower CI** | **Upper CI** |
| --- | --- | --- | --- | --- | --- |
| Intercept | -1.23 | 0.31 | -3.93 | -1.84 | -0.61 |
| Season-breeding | -0.37 | 0.17 | -2.20 | -0.70 | -0.04 |
| Season-summer | 0.97 | 0.18 | 5.54 | 0.63 | 1.31 |
| Season-fall | 1.23 | 0.20 | 6.31 | 0.85 | 1.62 |
| Sagebrush density bin 2 | -0.16 | 0.24 | -0.66 | -0.62 | 0.31 |
| Sagebrush density bin 3 | 1.38 | 0.26 | 5.27 | 0.87 | 1.90 |
| Sagebrush density bin 4 | 3.43 | 0.60 | 5.73 | 2.26 | 4.60 |
| Breeding*sagebrush density bin 2 | 1.14 | 0.34 | 3.39 | 0.48 | 1.80 |
| Summer*sagebrush density bin 2 | -0.64 | 0.26 | -2.46 | -1.15 | -0.13 |
| Fall*sagebrush density bin 2 | 1.01 | 0.37 | 2.76 | 0.29 | 1.73 |
| Breeding*sagebrush density bin 3 | -0.91 | 0.30 | -2.99 | -1.51 | -0.31 |
| Summer*sagebrush density bin 3 | -0.15 | 0.27 | -0.55 | -0.68 | 0.38 |
| Fall*sagebrush density bin 3 | -0.60 | 0.31 | -1.93 | -1.20 | 0.01 |
| Breeding*sagebrush density bin 4 | 0.55 | 0.62 | 0.89 | -0.66 | 1.76 |
| Summer*sagebrush density bin 4 | -1.41 | 0.61 | -2.31 | -2.60 | -0.21 |
| Fall*sagebrush density bin 4 | -1.75 | 0.69 | -2.55 | -3.09 | -0.41 |

**TABLE S12** Coefficients, *SE*s, *z*-values, and 95% confidence intervals (CI) from the best-performing linear mixed effects model (model weight 1.00) explaining selection of post-fire sagebrush/forb seeding, forb seeding, and herbicide treatment areas by greater sage-grouse in the Soda Wildfire area in southern Idaho and Oregon during 2016. Reference variable for season was breeding; sage-grouse were not tracked in winter of this year. Variables with CI ranges that did not overlap zero are important variables.

| **Variable** | **Coefficient** | ***SE*** | ***z*-value** | **Lower CI** | **Upper CI** |
| --- | --- | --- | --- | --- | --- |
| Intercept | 0.29 | 0.14 | 2.02 | 0.01 | 0.57 |
| Season-summer | 0.30 | 0.07 | 4.38 | 0.17 | 0.44 |
| Season-fall | 0.23 | 0.09 | 2.61 | 0.06 | 0.40 |
| Sagebrush/forb seeding-winter 2016 | -1.00 | 0.07 | -14.59 | -1.13 | -0.86 |
| Forb seeding-winter 2016 | 2.23 | 0.10 | 23.09 | 2.04 | 2.42 |
| Herbicide application-fall 2015 | 1.66 | 0.06 | 29.21 | 1.55 | 1.78 |

**TABLE S13** Coefficients, *SE*s, *z*-values, and 95% confidence intervals (CI) from the best-performing linear mixed effects model (model weight 1.00) explaining selection of post-fire sagebrush/forb seeding, forb seeding, and herbicide treatment areas by greater sage-grouse in the Soda Wildfire area in southern Idaho and Oregon during 2017. Reference variable for season was winter. Variables with CI ranges that did not overlap zero are important variables.

| **Variable** | **Coefficient** | ***SE*** | ***z*-value** | **Lower CI** | **Upper CI** |
| --- | --- | --- | --- | --- | --- |
| Intercept | 1.47 | 0.21 | 6.97 | 1.06 | 1.88 |
| Season-breeding | -0.77 | 0.11 | -7.20 | -0.98 | -0.56 |
| Season-summer | -0.35 | 0.10 | -3.44 | -0.55 | -0.15 |
| Season-fall | -0.33 | 0.12 | -2.82 | -0.56 | -0.10 |
| Sagebrush/forb seeding-winter 2016 | -0.34 | 0.06 | -5.80 | -0.45 | -0.23 |
| Sagebrush/forb seeding-winter 2017 | 3.27 | 0.09 | 34.97 | 3.09 | 3.45 |
| Forb seeding-winter 2016 | 0.99 | 0.08 | 12.18 | 0.83 | 1.15 |
| Forb seeding-winter 2017 | 0.73 | 0.07 | 10.56 | 0.60 | 0.87 |
| Herbicide application-fall 2016 | 0.49 | 0.05 | 9.90 | 0.39 | 0.59 |

**TABLE S14** Coefficients, *SE*s, *z*-values, and 95% confidence intervals (CI) from the best-performing linear mixed effects model (model weight 1.00) explaining selection of post-fire herbicide treatment areas by greater sage-grouse in the Soda Wildfire area in southern Idaho and Oregon during 2017. Reference variable for season was winter. Variables with CI ranges that did not overlap zero are important variables.

| **Variable** | **Coefficient** | ***SE*** | ***z*-value** | **Lower CI** | **Upper CI** |
| --- | --- | --- | --- | --- | --- |
| Intercept | 0.60 | 0.20 | 3.02 | 0.21 | 1.00 |
| Season-breeding | -0.94 | 0.11 | -8.72 | -1.15 | -0.73 |
| Season-summer | -0.70 | 0.10 | -6.72 | -0.90 | -0.49 |
| Season-fall | -0.36 | 0.12 | -2.94 | -0.59 | -0.12 |
| Herbicide application-fall 2015 | 2.96 | 0.05 | 58.50 | 2.86 | 3.05 |

**TABLE S15** Coefficients, *SE*s, *z*-values, and 95% confidence intervals (CI) from the best-performing linear mixed effects model (model weight 0.95) explaining selection of post-fire sagebrush/forb seeding, forb seeding, sagebrush/grass seeding, and drill seeding treatment areas by greater sage-grouse in the Soda Wildfire area in southern Idaho and Oregon during 2018. Reference variable for season was winter. Variables with CI ranges that did not overlap zero are important variables.

| **Variable** | **Coefficient** | ***SE*** | ***z*-value** | **Lower CI** | **Upper CI** |
| --- | --- | --- | --- | --- | --- |
| Intercept | 0.12 | 0.37 | 0.32 | -0.61 | 0.84 |
| Season-breeding | -0.75 | 0.14 | -5.40 | -1.02 | -0.48 |
| Season-summer | 0.35 | 0.15 | 2.36 | 0.06 | 0.64 |
| Season-fall | 0.81 | 0.18 | 4.49 | 0.46 | 1.17 |
| Sagebrush/forb seeding-winter 2016 | 0.44 | 0.08 | 5.58 | 0.29 | 0.60 |
| Sagebrush/forb seeding-winter 2017 | 2.94 | 0.09 | 31.25 | 2.76 | 3.13 |
| Forb seeding-winter 2016 | 0.24 | 0.06 | 4.05 | 0.13 | 0.36 |
| Forb seeding-winter 2017 | 0.21 | 0.07 | 2.84 | 0.06 | 0.35 |
| Sagebrush/grass seeding-winter 2018 | 0.81 | 0.08 | 10.38 | 0.66 | 0.96 |
| Drill seeding-fall 2015 | -1.70 | 0.15 | -11.31 | -2.00 | -1.41 |
| Slope | -1.90 | 0.09 | -21.82 | -2.07 | -1.73 |

**TABLE S16** Coefficients, *SE*s, *z*-values, and 95% confidence intervals (CI) from the best-performing linear mixed effects model (model weight 1.00) explaining selection of post-fire herbicide treatment areas by greater sage-grouse in the Soda Wildfire area in southern Idaho and Oregon during 2018. Reference variable for season was winter. Variables with CI ranges that did not overlap zero are important variables.

| **Variable** | **Coefficient** | ***SE*** | ***z*-value** | **Lower CI** | **Upper CI** |
| --- | --- | --- | --- | --- | --- |
| Intercept | 0.58 | 0.26 | 2.25 | 0.07 | 1.08 |
| Season-breeding | -0.98 | 0.15 | -6.73 | -1.26 | -0.69 |
| Season-summer | -0.49 | 0.15 | -3.22 | -0.79 | -0.19 |
| Season-fall | 0.17 | 0.18 | 0.94 | -0.18 | 0.52 |
| Herbicide application-fall 2015 | 4.57 | 0.08 | 54.25 | 4.40 | 4.73 |
| Herbicide application-fall 2016 | 1.37 | 0.09 | 15.84 | 1.20 | 1.54 |

**TABLE S17** Model-averaged coefficients, adjusted *SE*s, *z*-values, and 95% confidence intervals (CI) from the two best-performing linear mixed effects models (model weights ≥0.01) explaining selection of post-fire drill seeding treatment areas by greater sage-grouse in the Soda Wildfire area in southern Idaho and Oregon during 2016. Reference variable for season was breeding; sage-grouse were not tracked in winter of this year. Variables with CI ranges that did not overlap zero are important variables.

| **Variable** | **Coefficient** | **Adjusted *SE*** | ***z*-value** | **Lower CI** | **Upper CI** |
| --- | --- | --- | --- | --- | --- |
| Intercept | 0.07 | 0.21 | 0.34 | -0.34 | 0.49 |
| Season-summer | 0.18 | 0.09 | 2.03 | 0.01 | 0.35 |
| Season-fall | 0.17 | 0.10 | 1.72 | -0.02 | 0.36 |
| Drill seeding-fall 2015 | -1.84 | 0.10 | 18.73 | -2.03 | -1.65 |
| Slope | -1.70 | 0.06 | 27.80 | -1.82 | -1.58 |

**TABLE S18** Coefficients, *SE*s, *z*-values, and 95% confidence intervals (CI) from the best-performing linear mixed effects model (model weight 1.00) explaining selection of post-fire drill seeding treatment areas by greater sage-grouse in the Soda Wildfire area in southern Idaho and Oregon during 2017. Reference variable for season was winter. Variables with CI ranges that did not overlap zero are important variables.

| **Variable** | **Coefficient** | ***SE*** | ***z*-value** | **Lower CI** | **Upper CI** |
| --- | --- | --- | --- | --- | --- |
| Intercept | 0.82 | 0.20 | 4.12 | 0.43 | 1.20 |
| Season-breeding | -1.64 | 0.11 | -15.24 | -1.85 | -1.42 |
| Season-summer | -1.01 | 0.10 | -10.02 | -1.21 | -0.81 |
| Season-fall | -0.72 | 0.12 | -6.20 | -0.95 | -0.50 |
| Drill seeding-fall 2015 | -0.69 | 0.08 | -8.84 | -0.84 | -0.53 |
| Drill seeding-fall 2016 | 2.10 | 0.05 | 38.15 | 1.99 | 2.20 |
| Slope | -1.71 | 0.06 | -28.40 | -1.83 | -1.59 |

**TABLE S19** Coefficients, *SE*s, *z*-values, and 95% confidence intervals (CI) from the best-performing linear mixed effects model (model weight 1.00) explaining selection of post-fire drill seeding treatment areas by greater sage-grouse in the Soda Wildfire area in southern Idaho and Oregon during 2018. Reference variable for season was winter. Variables with CI ranges that did not overlap zero are important variables.

| **Variable** | **Coefficient** | ***SE*** | ***z*-value** | **Lower CI** | **Upper CI** |
| --- | --- | --- | --- | --- | --- |
| Intercept | -0.46 | 0.45 | -1.03 | -1.34 | 0.42 |
| Season-breeding | -0.45 | 0.13 | -3.63 | -0.70 | -0.21 |
| Season-summer | 0.84 | 0.14 | 6.22 | 0.58 | 1.11 |
| Season-fall | 0.43 | 0.16 | 2.59 | 0.10 | 0.75 |
| Drill seeding-fall 2016 | 2.71 | 0.07 | 41.29 | 2.58 | 2.83 |
| Drill seeding-fall 2017 | 4.42 | 0.27 | 16.17 | 3.89 | 4.96 |
| Slope | -0.64 | 0.07 | -9.23 | -0.78 | -0.50 |
